# Metabolic fluxes-oriented control of bioreactors: a novel approach to tune micro-aeration and substrate feeding in fermentations

**DOI:** 10.1186/s12934-019-1198-6

**Published:** 2019-09-04

**Authors:** Thiago José Barbosa Mesquita, Cíntia Regina Sargo, José Roberto Fuzer, Sheyla Alexandra Hidalgo Paredes, Roberto de Campos Giordano, Antonio Carlos Luperni Horta, Teresa Cristina Zangirolami

**Affiliations:** 1 Graduate Program of Chemical Engineering, Federal University of São Carlos (PPGEQ-UFSCar), Rodovia Washington Luís, Km 235, São Carlos, SP 13565-905 Brazil; 2 Graduate Program of Chemical Engineering-Institute of Chemistry, Federal University of Goiás (PPGEQ/IQ-UFG), Avenida Esperança, Campus Samambaia, Goiânia, GO 74690-900 Brazil

**Keywords:** Metabolic flux control, Micro-aeration, Bioreactor advanced control, Alcoholic fermentation, *Saccharomyces cerevisiae*

## Abstract

**Background:**

Fine-tuning the aeration for cultivations when oxygen-limited conditions are demanded (such as the production of vaccines, isobutanol, 2–3 butanediol, acetone, and bioethanol) is still a challenge in the area of bioreactor automation and advanced control. In this work, an innovative control strategy based on metabolic fluxes was implemented and evaluated in a case study: micro-aerated ethanol fermentation.

**Results:**

The experiments were carried out in fed-batch mode, using commercial *Saccharomyces cerevisiae*, defined medium, and glucose as carbon source. Simulations of a genome-scale metabolic model for *Saccharomyces cerevisiae* were used to identify the range of oxygen and substrate fluxes that would maximize ethanol fluxes. Oxygen supply and feed flow rate were manipulated to control oxygen and substrate fluxes, as well as the respiratory quotient (RQ). The performance of the controlled cultivation was compared to two other fermentation strategies: a conventional “Brazilian fuel-ethanol plant” fermentation and a strictly anaerobic fermentation (with ultra-pure nitrogen used as the inlet gas). The cultivation carried out under the proposed control strategy showed the best average volumetric ethanol productivity (7.0 g L^−1^ h^−1^), with a final ethanol concentration of 87 g L^−1^ and yield of 0.46 g_ethanol_ g_substrate_^−1^. The other fermentation strategies showed lower yields (close to 0.40 g_ethanol_ g_substrate_^−1^) and ethanol productivity around 4.0 g L^−1^ h^−1^.

**Conclusion:**

The control system based on fluxes was successfully implemented. The proposed approach could also be adapted to control several bioprocesses that require restrict aeration.

## Introduction

The performance of bioreactors depends on the control of several cultivation conditions (e.g.: pH, temperature, dissolved oxygen), in order to direct cell metabolism towards the improvement in yield, selectivity, and productivity of the target product. For example, the control of the oxygen supply to facultative microorganisms plays a key role in driving metabolic routes towards the production of biomass (favored under aerobic conditions) or the synthesis of some fermentative products (favored by low or zero oxygen availability) [1].

Conventional strategies in aerobic processes are essentially based on measurements of the dissolved oxygen concentration (DOC), which is then controlled by adjusting the stirrer speed and, occasionally, the composition of the gas supplied to the reactor (for instance, enrichment with pure oxygen) or the total gas flow rate [2, 3]. Some improvements have been made over time including more refined DOC controls through the use of mechanistic models [3] or neural networks [4], to mention a few.

However, the application of these strategies may be not effective under microaerobic conditions, because ordinary probes are not accurate at low dissolved oxygen tension [5]. Under oxygen-limited conditions, DOC < 1.5% [6], the control strategies previously mentioned may become unstable. Nevertheless, micro-aerobic conditions are required to produce many biotechnological products, including vaccines used against *H. influenzae*, *S. pnemoniae*, and *N. meningitidis* [7–9], and bioethanol production by *S. cerevisiae* [10], by *E. coli* [11], and by *P. stipitis* [12]. The production of advanced biofuels and bio-based chemical intermediates, including *n*-butanol [13], 2,3-butanediol [14] and acetone [15], important in the context of the emerging low carbon economy also relies on micro-aerobic cultivations. Bio-based processes can be used to convert agricultural residues’ biomass into industrially valuable products such as lactic acid [16] and bioethanol [17], serving as alternative routes to petrochemical products. Thus, the need for updated, robust, and low-cost control strategies for microaerobic fermentations in industrial bioreactors is likely to increase soon.

In the context of bioethanol production using *S. cerevisiae*, the catabolism of sugars is a key factor affecting process performance and is strongly influenced by oxygen availability. If the oxygen supplied is below a threshold value, glycerol will be produced, decreasing the ethanol yield [18]. On the other hand, if respiration is activated, greater substrate fluxes will be directed towards biomass formation [18], also leading to reduced ethanol yields. Providing a suitable oxygen flux seems important for 2nd generation ethanol production from xylose because it helps to relieve observed redox imbalances through cofactor regeneration [19] and to maintain sufficient amino acids and protein synthesis, necessary for growth [20, 21]. Furthermore, micro-aeration helps to preserve cell viability, hence affecting ethanol productivity [10, 22].

Despite the importance of the control of micro-aerobic conditions in fermentations, there are not many studies addressing this topic in the literature. The use of a constant (sparged or headspace) air flow, without any control, to cultivate *S. cerevisiae* under oxygen limitation was reported in a couple of works [10, 22, 23]. Even RQ-based control, extensively studied to adjust feeding supply for aerobic *S. cerevisiae* cultures [24, 25], was little explored to set up micro-aeration control strategies. In fact, this approach was applied to continuous fermentations using *S. cerevisiae* and consisted in the manipulation of the inlet gas stream composition to maintain RQ at the desired level and enforce microaerobic conditions [26]. Easily estimated from off-gas measurements and available on-line, RQ provides valuable information about *S. cerevisiae* respiratory activity [24, 25]. Furthermore, when RQ is integrated with other inputs within a suitable framework, a more complete control system, able to tune both oxygen and carbon source supplies, can be implemented.

In this context, genome-scale metabolic models (GSMs) can also be employed for bioreactor control. They are a source of in silico data reproducing the cell metabolism. With these models, it is possible to estimate intracellular and extracellular fluxes of metabolites at steady-state for different environmental conditions, considering the inlet fluxes of substrate and oxygen—which in fact drive the metabolic reactions occurring within the cell. *Saccharomyces cerevisiae*, in particular, has been extensively studied, with at least 12 GSMs published ever since 2003 [27]. *S. cerevisiae* GSMs have been employed with satisfactory results to improve the understanding of yeast physiology, as well as to predict targets for metabolic engineering in order to overproduce chemicals such as succinic acid [28], fumaric acid [29], bioethanol [30], 2,3-butanediol [31] and l-tyrosine [32]. Still, the application of metabolic models is not restricted to understanding cell pathway networks or improving them. Indeed, metabolic models may be powerful tools for process improvement and for bioreactor’s control. With this kind of information, estimated metabolic fluxes can be used to develop cultivation strategies better tuned with the cell actual metabolism.

GSMs are stoichiometric models, which assume steady-state behavior. Usually, this is not the case in real fermentations, especially in industrial scale, but the information concerning metabolic fluxes may delimit regions of operation for the reactor. Following this concept, the GSMs output can be understood as a pseudo-stationary response to the environmental conditions observed during the process. These estimates of substrate/product fluxes may provide useful information for the control system, even though the bioreactor does not operate at steady-state.

An innovative bioreactor control system is herein reported, based on metabolic fluxes estimated using a GSM. This new approach is evaluated using the production of bioethanol under microaerobic conditions as a case study. Indeed, an important problem in industrial fermentations is to increase bioethanol production at the expense of biomass and glycerol, and this was the focus of this work. A novel supervisory system is also presented, which combines advanced strategies for bioprocess control, on-line data acquisition, and a flux-oriented control model.

## Results

The development of the metabolic flux-oriented control involves several steps including GSM simulations, selection of suitable simulated data, and their integration with model equations to configure the control system. Additionally, it is important to assess the control performance by applying it to a real process. In the present work, ethanol production in *S. cerevisiae* fermentation operated in fed-batch mode was chosen as a case study to test the flux-based micro-aeration control (FMC).

**Fig. 1:**
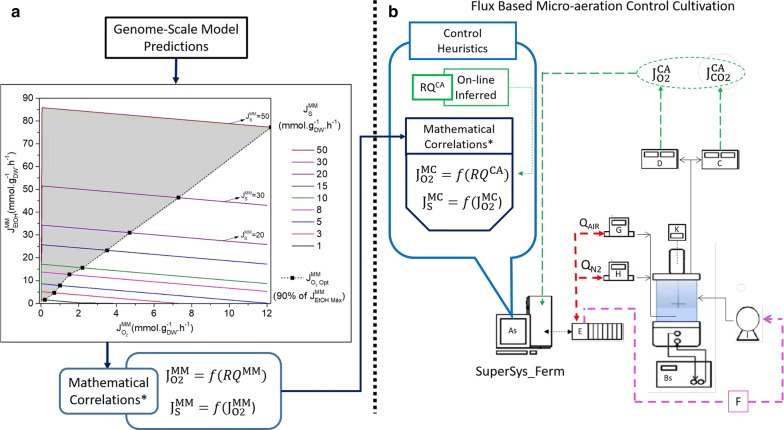
Schematic representation of the proposed control strategy for the Flux-based Micro-aerated control fermentation (FMC). **a** Simulated ethanol fluxes using the genome-scale metabolic model (GSM) of *S. cerevisiae* iND750 for different glucose and oxygen fluxes as inputs. The gray shaded area in A represents the maximum GSM simulated ${\text{J}}_{\text{EtOH}}^{\text{MM}}$ region. ${\text{J}}_{{{\text{O}}_{2} }}^{\text{MM}}$—simulated oxygen flux; ${\text{J}}_{\text{EtOH}}^{\text{MM}}$—simulated ethanol flux; ${\text{J}}_{\text{S}}^{\text{MM}}$—simulated substrate flux. **b** FMC control framework, with RQ as the controlled variable, and both Q_AIR_ and F as manipulated variables. Fluxes: ${\text{J}}_{\text{S}}^{\text{MC}}$ and ${\text{J}}_{{{\text{O}}_{2} }}^{\text{MC}}$—mathematical correlation substrate and O_2_ flux; ${\text{J}}_{{{\text{O}}_{2} }}^{\text{CA}}$ and ${\text{J}}_{{{\text{O}}_{2} }}^{\text{CA}}$—O_2_ and CO_2_ fluxes calculated on-line by the control algorithm; ${\text{RQ}}_{{}}^{\text{CA}}$—respiratory quotient calculated on-line. Equipment: As-computational monitoring and supervision system SuperSys_Ferm; Bs—Bath; C—CO_2_ analyzer; D—O_2_ analyzer; E—cFP-FieldPoint; K—Impeller speed controller; G—Air flow controller; H—Nitrogen flow controller; I—feed pump. Dashed lines—information. Control loops: control of oxygen consumption (Air flow rate—Q_AIR_, Eq. B2.3; Nitrogen flow rate—iQ_N2_) (in red); control of substrate consumption (Feed flow rate—F, Eq. B2.1) (in lilac); Controlled variable (RQ) (in green). Some lines of acquisition and communication with the field point are omitted. All fluxes in mmol g_DW_^−1^ h^−1^. *Mathematical correlations in Results Metabolic Models Simulations and Correlations (Eqs. 1 and 2, respectively)

Initially, simulations using the genome-scale metabolic model of *S. cerevisiae* iND750 [33] and the Optflux 3.2.7 software [34] were run. From this in silico study, simulated metabolic fluxes (J_i_^MM^) for different species (substrate, ethanol, CO_2_, O_2_, etc.) were collected and used to generate mathematical correlations (MC), which in turn provided estimations of substrate $\left( {{\text{J}}_{\text{S}}^{\text{MC}} } \right)$ and oxygen $\left( { {\text{J}}_{{{\text{O}}_{2} }}^{\text{MC}} } \right)$ fluxes, to be used by the control algorithm (Fig. 1a). Results concerning the use of the metabolic model simulations to obtain J^MM^, as well as the mathematical correlations to predict J^MC^, are presented in the following section.

In the next step, the controller was set up. In brief, from a set of on-line (or at-line) inputs (including volume, N_2_ flow rate, cell mass, temperature, pressure and outlet gas composition), combined with equations derived from mass balances, the selected outputs, inlet O_2_ mol fraction (${\text{y}}_{{{\text{O}}_{ 2} {\text{ in}}}}$) and control action fluxes $\left( {{\text{J}}_{{{\text{O}}_{2} }}^{\text{CA}} ,{\text{J}}_{{{\text{CO}}_{2} }}^{\text{CA}} } \right)$, were estimated. Control action fluxes were used as inputs to the RQ loop, which delivered ${\text{J}}_{\text{S}}^{\text{MC}}$ and ${\text{J}}_{{{\text{O}}_{2} }}^{\text{MC}}$. Together with the other inputs, ${\text{J}}_{{{\text{O}}_{2} }}^{\text{MC}}$ was used to update the air flow rate (Q_air_) whereas ${\text{J}}_{\text{S}}^{\text{MC}}$ updated the volumetric flow rate of feeding medium (F) (Fig. 1b).

The control performance is also described below. The detailed description of the control strategy is available in the methods and the main equations and control logic are given at Boxes 2 and 3, respectively (the equations derivations are available in Additional file 1). The agreement between experimental and model estimated fluxes is also used herein to evaluate the proposed control strategy. To do so, experimental fluxes (${\text{J}}_{\text{i}}^{\text{Exp}}$) for ethanol, substrate, and biomass were calculated from off-line data using component mass balances (as depicted in Box 1) and compared to the corresponding J_i_^MM^ fluxes.

Finally, the performance of the fermentation carried out under the Flux-based Micro-aeration Control (FMC) was compared to other fermentation strategies: a conventional fermentation strategy, used in Brazilian Bioethanol Plants (BBP), and another cultivation carried out under strictly anaerobic conditions (SAC).

### Metabolic model simulations and correlations implemented in the control algorithm

**Fig. 2:**
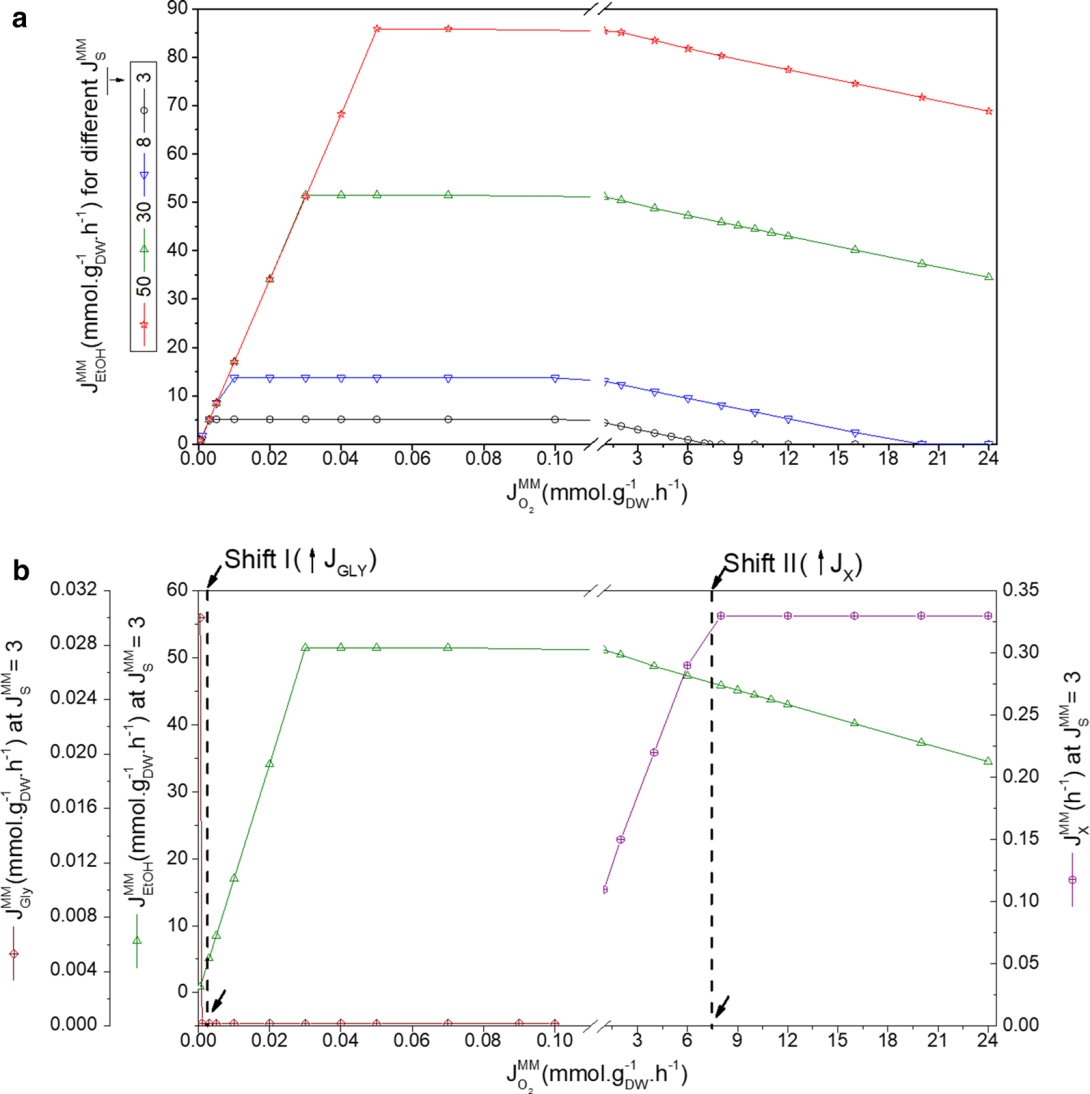
Metabolic shifts predicted by the GSM for different oxygen and substrate fluxes. **a** Metabolic fluxes predicted by the genome-scale metabolic model (GSM) of *S. cerevisiae* iND750 [33]. **b** An example of the metabolic shifts predicted by the GSM, for a glucose flux of 3 mmol g_DW_^−1^ h^−1^. Shift I: increase of ${\text{J}}_{\text{Gly}}^{\text{MM}}$ and decrease of ${\text{J}}_{\text{EtOH}}^{\text{MM}}$; Shift II: increase of ${\text{J}}_{\text{X}}^{\text{MM}}$ and decrease of ${\text{J}}_{\text{EtOH}}^{\text{MM}}$

To select the most suitable oxygen levels to be implemented by the proposed flux-based micro-aeration control, a more detailed view of the influence of oxygen on yeast metabolism is required. Several simulations of the genome-scale metabolic model (GSM) of *S. cerevisiae* iND750 [33] were run, using the software Optflux 3.2.7 [34]. Different combinations of inlet fluxes, ${\text{J}}_{\text{S}}^{\text{MM}}$ and ${\text{J}}_{{{\text{O}}_{2} }}^{\text{MM}}$, were used as inputs to Optflux, and the corresponding output fluxes for ethanol, biomass, CO_2_, and glycerol were generated for each simulated condition (Fig. 2a).

Figure 2b shows the metabolic shifts that occur when the flux of oxygen is increased (see also Additional file 1: Table S1), for a specified flux of substrate (in this case, for example, the substrate flux was 3 mmol g_DW_^−1^ h^−1^). A first shift occurs at a minimum critical value of ${\text{J}}_{{{\text{O}}_2}}^{\text{MM}}$ (Fig. 2b, Shift I—condition: ${\text{J}}_{{{\text{O}}_{2} }}^{\text{MM}} < 7{\text{E}}^{ - 4} * {\text{J}}_{\text{S}}^{\text{MM}}$). Simulations with smaller values than this critical ${\text{J}}_{{{\text{O}}_{2} }}^{\text{MM}}$ resulted in a decrease in ethanol fluxes, also triggering an increase of the fluxes of glycerol and other metabolites. As ${\text{J}}_{{{\text{O}}_2}}^{\text{MM}}$ is increased, a second metabolic shift is predicted by the model: substrate fluxes were directed towards biomass formation and ${\text{J}}_{\text{EtOH}}^{\text{MM}}$ decreased (Fig. 2b, Shift II—condition: ${\text{J}}_{{{\text{O}}_{2} }}^{\text{MM}} > 2.44*{\text{J}}_{\text{S}}^{\text{MM}}$).

The responses of the metabolic model were consistent with the changes in *S. cerevisiae* metabolism that were expected under the influence of different fluxes of oxygen: biomass formation was favored at higher oxygen fluxes, while glycerol production was intensified at low oxygen fluxes, as already reported [35]. From the results displayed in Fig. 2, it is possible to define a range for operation of the reactor, aiming at maximizing ethanol production: the grey area seen in Fig. 1a, constrained by the glycerol shift (Shift I). However, operation close to Shift I would be too unstable to be considered for control purposes. So, sub-optimal ${\text{J}}_{\text{S}}^{\text{MM}}$ and ${\text{J}}_{{{\text{O}}_{2} }}^{\text{MM}}$ values were used to set up the proposed control strategy, corresponding to approximately 90% of the maximum predicted ${\text{J}}_{\text{EtOH}}^{\text{MM}}$ (the dotted line in Fig. 1a, constraining the right side of the operational region).

In order to implement the information mapped through GSM simulations (Figs. 1a, 2a) in the control algorithm (further details in Methods Control Algorithm Design), a linear correlation between ${\text{J}}_{\text{S}}^{\text{MM}}$ and ${\text{J}}_{{{\text{O}}_{2} }}^{\text{MM}}$ was fitted, see Eq. 1 and Additional file 1: Figure S1. The set of fluxes that provide 85–100% of the maximum flux of ethanol was used for this purpose.

1 $${\text{J}}_{\text{S}}^{\text{MC}} = \left( {3.9 \pm 0.2 } \right) * {\text{J}}_{{{\text{O}}_2}}^{\text{MC}} + \left( {1.2 \pm 0.8} \right)$$

Equation 1 was used to estimate ${\text{J}}_{\text{S}}^{\text{MC}}$, given the flux of oxygen, which was provided by a second correlation, a hyperbola, between RQ^MM^ and ${\text{J}}_{{{\text{O}}_{2} }}^{\text{MM}}$ (Additional file 1: Figure S2), see Eq. 2. The inlet air flow rate (Q_AIR_) was updated using Eqs. 2 and B2.3 (as described in Box 2). Higher ethanol fluxes were achieved for RQ values in the range 7–8 (available in Additional file 1: Figure S2) and, therefore, the RQ control loop was set to operate within this range.

2 $${\text{J}}_{{{\text{O}}_{2} }}^{\text{MC}} = \frac{{(0.37 \pm 0.06) * {\text{RQ}}^{\text{CA}} }}{{{\text{RQ}}^{\text{CA}} - (7.04 \pm 0.04)}}$$

These two correlations, which absorbed the information provided by the metabolic model, were at the core of the FMC algorithm. The supervisory program used these correlations to estimate on-line the metabolic fluxes ${\text{J}}_{{{\text{O}}_2}}^{\text{MC}}$ and ${\text{J}}_{\text{S}}^{\text{MC}}$. Then, these fluxes were converted into air flow rate (Q_AIR_) and fresh medium feed rate (F), respectively (by the series of equations given in Boxes 1 and 2). Both were manipulated to control RQ within the range 7.2–8.4. The overall performance of the FMC fermentation strategy is presented in the next section.

### Performance of the flux-based control (FMC)

Using the control logic previously summarized (and further details in the methods and Boxes 1, 2 and 3), implemented within the LabView^®^ (version 2015) framework, experiments were carried out in fed-batch mode, using defined medium containing glucose as the sole carbon source. Glucose, biomass, ethanol and glycerol profiles for the reactor operating under FMC can be seen in Figure SM3 (Additional file 1). The cultivation strategy was split into three stages: (i) a batch, aerobic phase (until 2.6 h) to increase biomass concentration; (ii) the fed-batch phase carried out under micro-aeration conditions implemented by the proposed control strategy to supply glucose and O_2_ (from 2.6 to 10.6 h); (iii) a final batch phase (from 10.6 to 12.6 h), started when glucose feeding was stopped (at 10.6 h), still carried out under micro-aeration conditions implemented by the proposed control strategy for O_2_ supply.

**Box 1.**
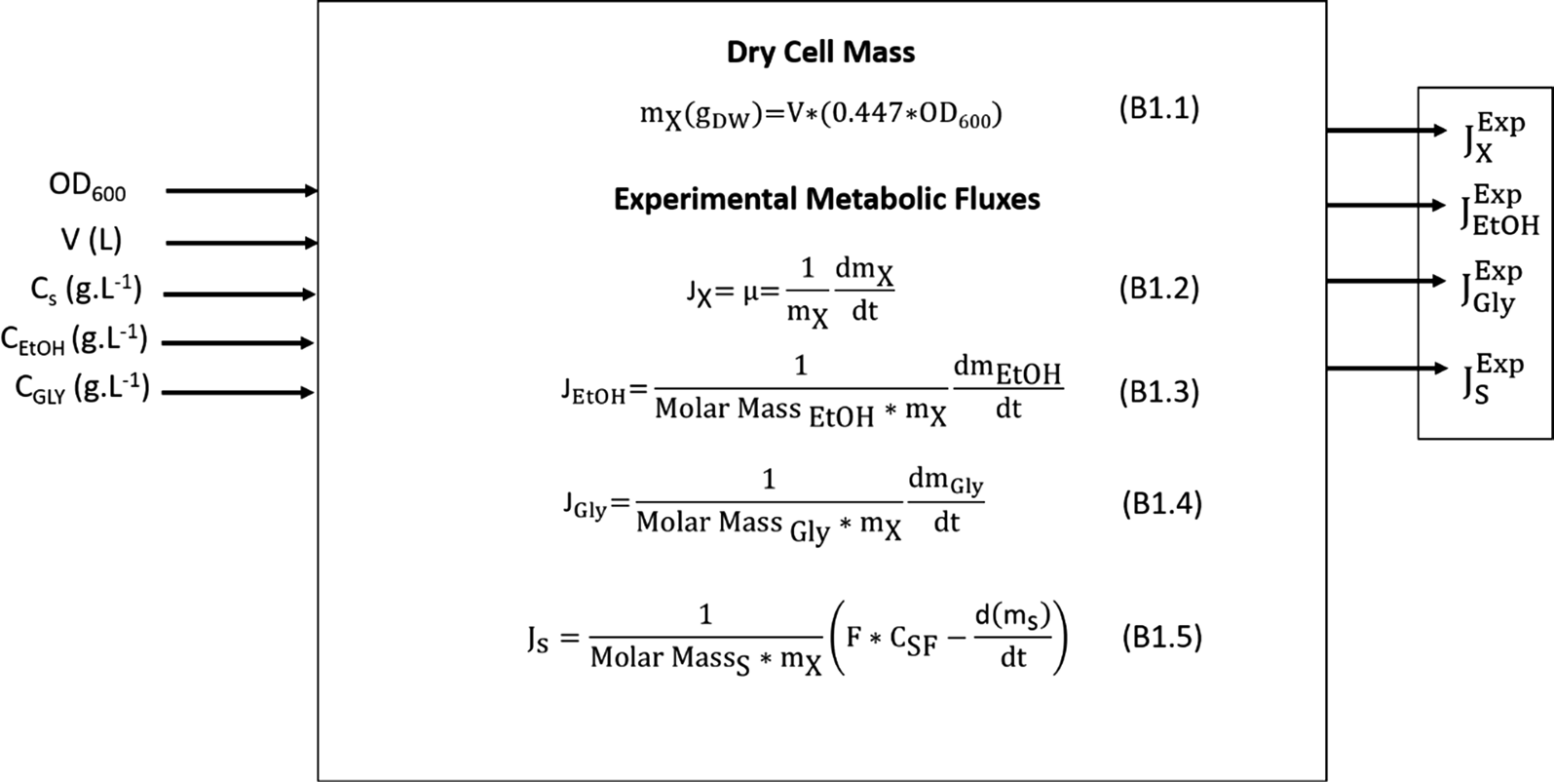
Calculation procedures for experimental fluxes. The values for constants and other conditions are: ${\text{y}}_{{{\text{CO}}_{ 2} , {\text{ in}}}}$ = 0.04 * 10^−2^; T_in_ = 294.25 K; P_out_ = 1 atm; R = 0.08206*10^−3^ L atm mmol^−1^ K^−1^. The variables ${\text{y}}_{{{\text{CO}}_{ 2} , {\text{ out}}}}$, ${\text{y}}_{{{\text{O}}_{ 2} , {\text{ out}}}}$ and T_out_ were measured on-line and accessed through the supervisory software. ${\text{J}}_{\text{s}}^{\text{Exp}}$, ${\text{J}}_{\text{EtOH}}^{\text{Exp}}$, ${\text{J}}_{\text{Gly}}^{\text{Exp}}$, ${\text{J}}_{\text{X}}^{\text{Exp}}$ are expressed in mmol g_DW_^−1^ h^−1^ and were obtained from off-line data. Volume (V, in L) was estimated at-line after integration of feed flow rate and subtraction of medium withdrawals. Mass and mole data are expressed in g and mmol, respectively

**Box 2.**
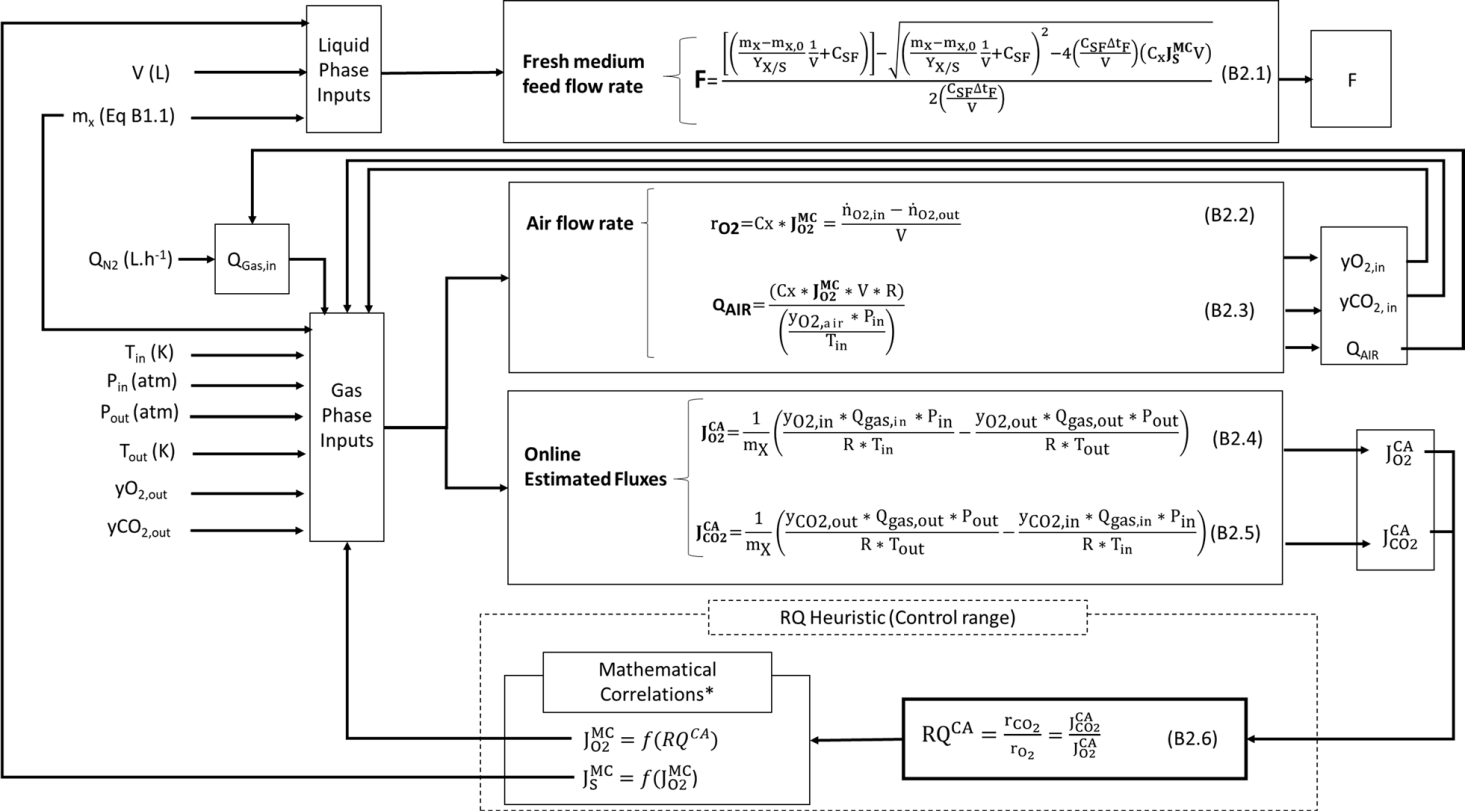
Flux-based micro-aerated control (FMC): equations for updating fresh medium feeding and air flow rates. The values for constants and other conditions are: ${\text{y}}_{{{\text{CO}}_{ 2} , {\text{ N}}_{ 2} }}$ = 0.01*10^−2^; T_in_ = 294.25 K; P_out_ = 1 atm; R = 0.08206*10^−3^ L atm mmol^−1^ K^−1^. Q_gas,out_, ${\text{y}}_{{{\text{CO}}_{ 2} , {\text{ in}}}}$, and ${\text{y}}_{{{\text{O}}_{ 2} , {\text{ in}}}}$ were estimated through mass balance. The variables ${\text{y}}_{{{\text{CO}}_{ 2} , {\text{ out}}}}$, ${\text{y}}_{{{\text{O}}_{ 2} , {\text{ out}}}}$ and T_out_ were measured on-line and accessed through the supervisory software. ${\text{J}}_{\text{s}}^{\text{MC}}$, ${\text{J}}_{{{\text{O}}_2}}^{\text{MC}}$, ${\text{J}}_{{{\text{O}}_{2} }}^{\text{CA}}$, ${\text{J}}_{{{\text{CO}}_{2} }}^{\text{CA}}$ are expressed in mmol g_DW_^−1^ h^−1^. Q_N2_, Q_AIR_ and F were manipulated and are expressed in L h^−1^. *Mathematical correlations in Results Metabolic Models Simulations and Correlations (Eqs. 1 and 2, respectively)

**Box 3.**
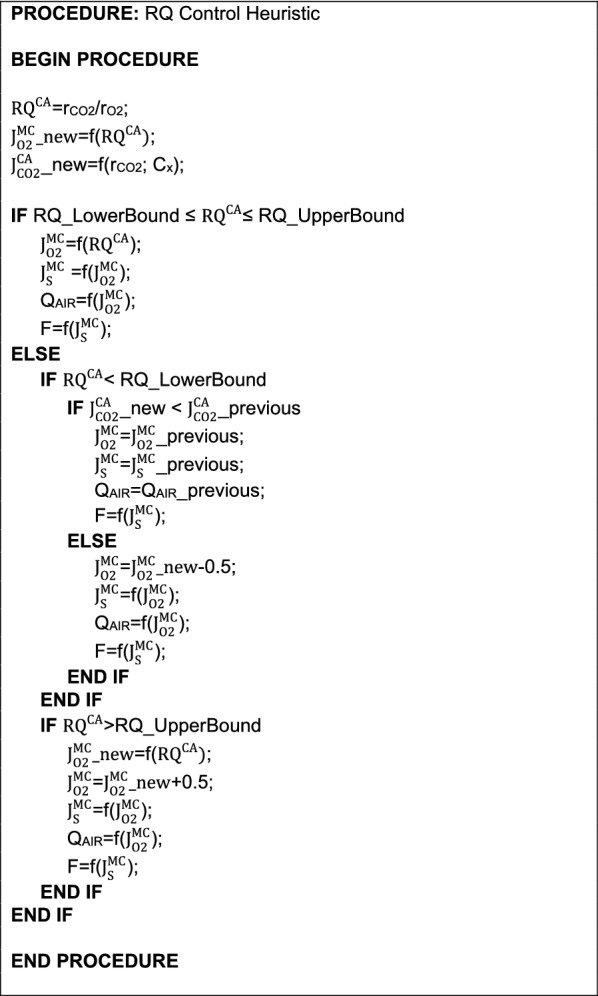
RQ control heuristics and framework

**Fig. 3:**
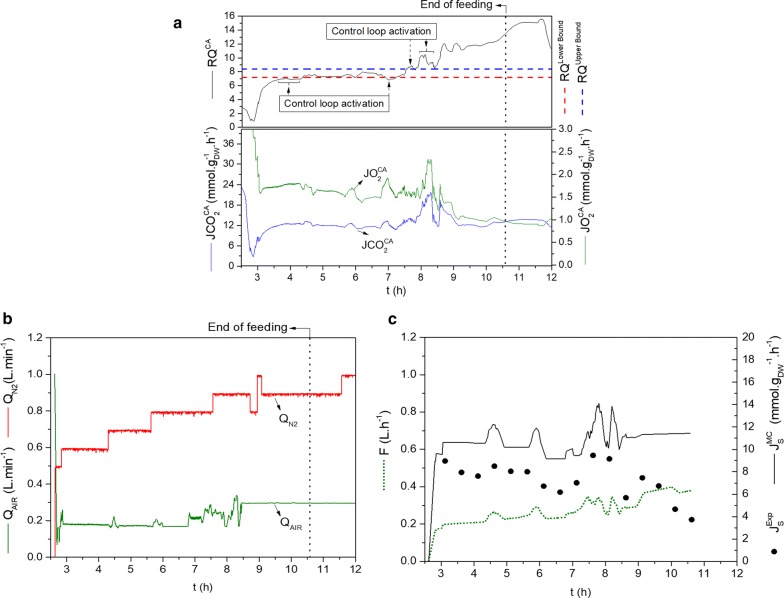
Overall performance of the flux-based Micro-aerated Control. **a** Respiratory quotient (RQ), oxygen flux (${\text{J}}_{{{\text{O}}_{2} }}^{\text{CA}}$), and carbon dioxide flux $\left( {{\text{J}}_{{{\text{CO}}_{2} }}^{\text{CA}} } \right)$ in FMC fermentation. The arrows indicate the activation of the RQ loop, which was deactivated when RQ returned to the set boundaries (lower boundary: red dashed line, upper boundary: blue dashed line) or when DOT $\ge$ 4.5%. **b** Q_N2_ and Q_AIR_ flow rates during the feeding phase and up to the end of the cultivation. **c** Fresh medium feed flow rate and substrate flux $\left( {{\text{J}}_{\text{S}}^{\text{CA}} } \right)$ in FMC fermentation

The overall performance of the controller, particularly in sustaining a micro-aerated environment, can be assessed from ${\text{J}}_{{{\text{O}}_{2} }}^{\text{CA}}$, ${\text{J}}_{{{\text{CO}}_{2} }}^{\text{CA}}$ and ${\text{RQ}}^{\text{CA}}$ data (Fig. 3a) as well as from the profiles of the manipulated air and N_2_ flow rates (Fig. 3b).

After 2.6 h the dissolved oxygen was set to zero (Additional file 1: Figure S3) by injecting N_2_, and the control action started. The gas phase control action consisted of correcting the air inlet flow to keep RQ between 7.2 and 8.4. Within this control range, the inlet air flow was updated using Eq. 2 and the other equations are given at Box 2. Q_N2_ was increased in steps during the cultivation to keep the outlet CO_2_ mol fraction within the gas analyzer maximum detection limit (20% CO_2_). The micro-aeration control remained active until all glucose was consumed at the end of the 3^rd^ stage of the FMC experiment in order to get a full picture of its performance.

The controlled variable (RQ^CA^) (Fig. 3a) followed the proposed control logic throughout the experiment. RQ^CA^ remained close to the lower boundary of 7.2 until 6.8 h. At this moment, RQ^CA^ values presented oscillations, and subsequently, they remained above the upper RQ^CA^ boundary of 8.4 mol CO_2_ mol O_2_^−1^. The observed oscillations matched the increase in the dissolved oxygen tension (Additional file 1: Figure S3).

A reduction of the cell fermentative capability, probably caused by ethanol inhibition or viability loss, as discussed later, may have contributed to decreasing the specific growth rate (or biomass flux J_X_) and, consequently, the oxygen uptake flux $\left( {{\text{J}}_{{{\text{O}}_{2} }}^{\text{CA}} } \right)$, as well (Fig. 3a). Once RQ is calculated by the ratio $\left( {{\text{J}}_{{{\text{CO}}_{2} }}^{\text{CA}} *{\text{J}}_{{{\text{O}}_{2} }}^{{{\text{CA}}^{ - 1} }} } \right)$ (Box 2, Eq. B2.6), low ${\text{J}}_{{{\text{O}}_{2} }}^{\text{CA}}$ values would result in the increasing RQ pattern observed from 8.5 to 11.5 h of FMC fermentation (Fig. 3a). The change in RQ trajectory in the last 30 min of experiment could be attributed to the activation of the respiratory metabolism, once Additional file 1: Figure S3 showed an increase in the biomass concentration close to the ending of the cultivation.

The control loop, which slightly increased or decreased the air flow (as can be seen in Box 3 and further explained in Methods Control Adjustments and Heuristics), was activated only for RQ values below or above the boundaries (shown as dotted lines in Fig. 3a). Thus, 75% of control action time was solely based on the correlation given by Eq. 2 (Boxes 2 and 3), which is directly derived from the metabolic model simulations. After 8.5 h, the control loop was deactivated because the dissolved oxygen tension remained over 4.5%. When it happens, the inlet air flow was kept at the last calculated value by the control code.

Similarly, the overall performance of the controller on supplying glucose can be assessed from ${\text{J}}_{\text{S}}^{\text{MC}}$ data as well, as from the profile of the manipulated fresh medium flow rate (Fig. 3c). The feeding stage lasted up to 10.6 h when all fresh medium prepared (~ 2.0 L) was added to the bioreactor (Fig. 3c, Additional file 1: Figure S3). The fresh medium flow (F) (Fig. 3c) was updated according to ${\text{J}}_{\text{S}}^{\text{MC}}$ values given by Eq. 1, using the Optical Density (OD_600 nm_) values obtained at-line, and the on-line calculated volume (V) (Box 2).

Regarding the manipulated variables within the control framework, the manipulation of the air flow rate worked efficiently (Fig. 3b) until the observed oscillations. From this moment on, the air flow rate was fixed at the last updated value by the controller. The fresh medium flow rate (F) was calculated by Eq. B.2.1 (Box 2) using the overestimated values for ${\text{J}}_{\text{S}}^{\text{MC}}$ (Fig. 3c), which were higher than the actual substrate consumption flux (represented by the ${\text{J}}_{\text{S}}^{\text{Exp}}$ at Fig. 3c) throughout the feeding phase. Consequently, F was overestimated too, and substrate accumulated in the broth (Additional file 1: Figure S3). The overestimation of ${\text{J}}_{\text{S}}^{\text{MC}}$ is due to the use of overestimated values of ${\text{J}}_{{{\text{O}}_{2} }}^{\text{MC}}$ in Eq. (1). Figure 3c shows that misfit between ${\text{J}}_{\text{S}}^{\text{MC}}$ and ${\text{J}}_{\text{S}}^{\text{Exp}}$ increased after 8.6 h of fermentation because, at this moment, the air flow rate was set to a fixed value. Consequently, ${\text{J}}_{{{\text{O}}_{2} }}^{\text{MC}}$ and ${\text{J}}_{\text{S}}^{\text{MC}}$ started to follow constant patterns, too. The intensification of ethanol inhibition after 8.5 h contributed to a further decrease the actual ${\text{J}}_{\text{S}}^{\text{Exp}}$ (Fig. 3c) and, in turn, aggravated glucose accumulation (Additional file 1: Figure S3).

### Comparison of experimental and simulated fluxes for ethanol, biomass and substrate in FMC fermentation

**Fig. 4:**
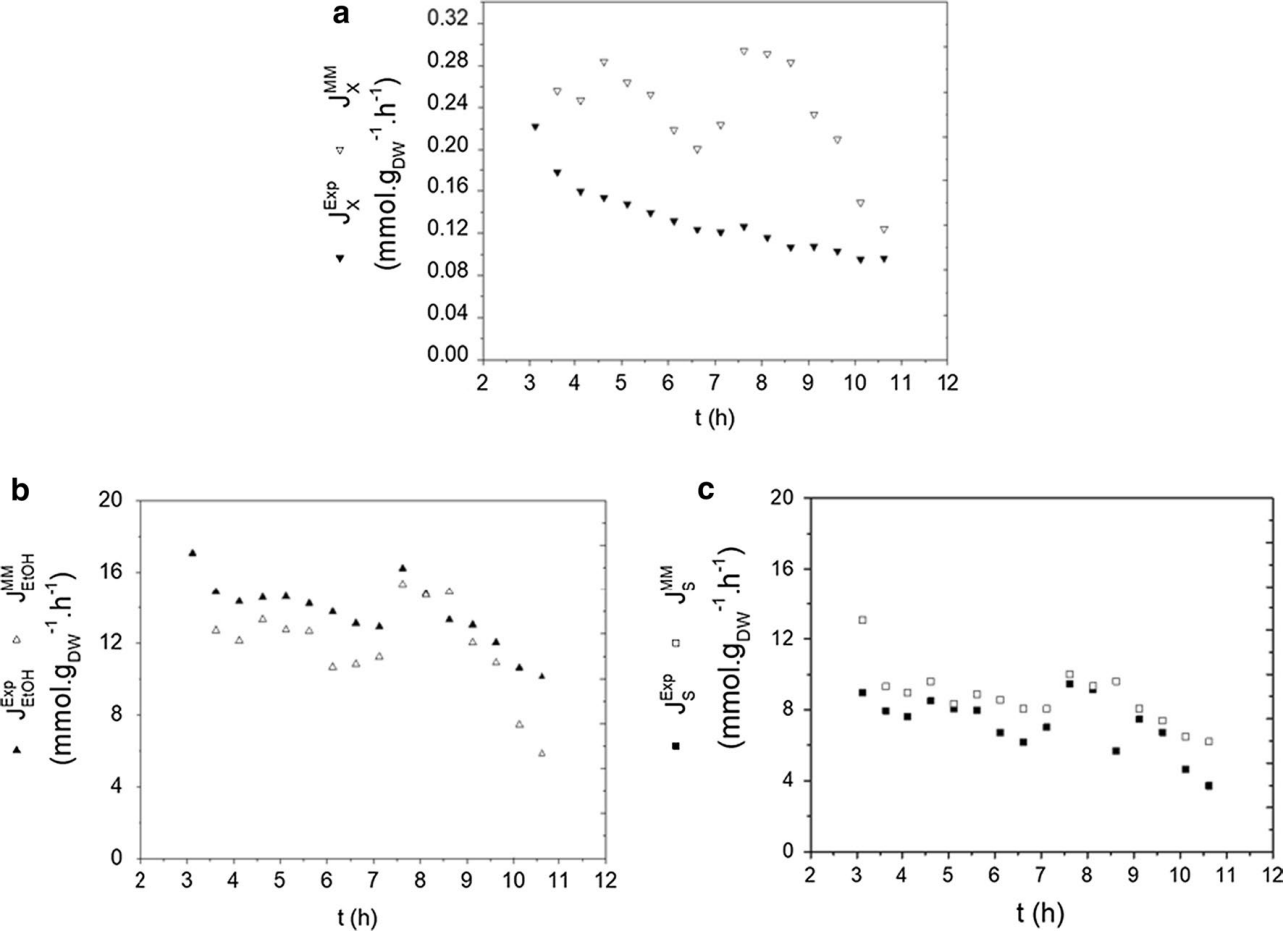
Simulated and experimental fluxes for FMC fermentation during the fed-batch phase. Metabolic fluxes of **a** biomass, **b** ethanol and **c** substrate

As previously discussed, the performance of the proposed control strategy depends on how well the metabolic model predictions reproduce the actual cell behavior. Thus, after the FMC fermentation was performed, off-line data describing the trends in the concentrations of biomass, glucose, and ethanol became available and they were used to estimate the experimental fluxes by using component mass balances (according to equations in Box 1 and further detailed in Methods Experimental Flux and Cultivation Parameters Calculation). Then, the experimental fluxes ${\text{J}}_{\text{X}}^{\text{Exp}}$, ${\text{J}}_{\text{EtOH}}^{\text{Exp}}$,${\text{J}}_{\text{S}}^{\text{Exp}}$ could be compared to the corresponding fluxes estimated by the metabolic model ${\text{J}}_{\text{X}}^{\text{MM}}$ ${\text{J}}_{\text{EtOH}}^{\text{MM}}$, and ${\text{J}}_{\text{S}}^{\text{MM}}$ (Fig. 4). To enable this comparison, the metabolic model fluxes ${\text{J}}_{\text{X}}^{\text{MM}}$ and ${\text{J}}_{\text{EtOH}}^{\text{MM}}$ were calculated by using the same values of ${\text{J}}_{{{\text{O}}_2}}^{\text{Exp}}$ and ${\text{J}}_{\text{S}}^{\text{Exp}}$ as inputs for the Optflux simulations. Whereas for ${\text{J}}_{\text{S}}^{\text{MM}}$, the values of ${\text{J}}_{{{\text{O}}_{2} }}^{\text{Exp}}$ and ${\text{J}}_{\text{EtOH}}^{\text{Exp}}$ were used as inputs for these simulations.

The experimental and simulated fluxes for ethanol and substrate showed similar profiles (Fig. 4b, c), whereas the experimental biomass fluxes were always lower than the corresponding metabolic fluxes (Fig. 4a). This overestimation of biomass fluxes in the simulation of metabolic models is inherent to the use of the objective function that maximizes growth. Biomass fluxes generated from metabolic model simulations for this objective function, in fact, represent the theoretical upper limit for the maximum specific growth rate that could be attained by the cells under the model constraints. So, as expected, in vivo biomass fluxes remained below these maximum theoretical values. As further detailed at the Methods Metabolic Model Simulations, the maximization of growth was chosen as the objective function because it provides a better emulation of the actual yeast cell metabolism than other options, such as maximization of ethanol flux.

The decrease in ${\text{J}}_{\text{X}}^{\text{Exp}}$, ${\text{J}}_{\text{EtOH}}^{\text{Exp}}$, and ${\text{J}}_{\text{S}}^{\text{Exp}}$ as ethanol concentrations increased (Fig. 4a–c) is another notable trend. The observed decreasing pattern for cells and substrate uptake was probably related to ethanol inhibition, which is intensified at ethanol concentrations above 0.75 M [36, 37]. This threshold value for the ethanol concentration that triggers inhibition can vary according to the strain and cultivation conditions [37]. Besides experimental fluxes, the fluxes ${\text{J}}_{\text{X}}^{\text{MM}}$,${\text{J}}_{\text{EtOH}}^{\text{MM}}$ and ${\text{J}}_{\text{S}}^{\text{MM}}$ estimated from model simulations also showed decreasing trends at high ethanol concentrations (Fig. 4a–c), because smaller values of ${\text{J}}_{{{\text{O}}_{2} }}^{\text{Exp}}$ were observed at this cultivation condition, and provided as inputs to Optflux.

Regarding glycerol production, there was an accumulation of 9 g L^−1^ at the end of the fermentation (Additional file 1: Figure S3). Experimental glycerol fluxes decreased from 1.4 to 0.7 mmol g_DW_^−1^ h^−1^ from the beginning to the end of the fermentation (data not shown). This production is higher than the values predicted by the metabolic model, which would vary from 0 to 0.12 mmol g_DW_^−1^ h^−1^ (data not shown) for the values of ${\text{J}}_{{{\text{O}}_{2} }}^{\text{Exp}}$ and ${\text{J}}_{\text{S}}^{\text{Exp}}$ used as inputs. Thus, the metabolic model iND750 developed by Duarte and coworkers (2004) seems to reproduce cell metabolism in terms of ethanol production and substrate uptake, under the studied conditions, but it failed to describe glycerol production. Nevertheless, it should be once again stressed that GSMs are stoichiometric models, and their results must be seen as indicators of trends when transient cultivations are processed in real bioreactors. Moreover, the GSMs predictions were inputs for a control action that provided overall performance indices superior to conventional fermentation strategies, as discussed in the next section. Besides, the control variable, RQ, could also be effectively controlled adequately during most of the fed-batch, as shown in Fig. 3a. And the insights provided by the simulations were very important to define the bioreactor operational strategy.

### Performance of different cultivation strategies

In the previous topics, the flux-based micro-aeration control (FMC) was detailed and its implementation was described. However, the embracement of the FMC strategy in real life cultivations will depend on its ethanol productivity and yield, when compared to other fermentation strategies. So, two additional reference cultivations were performed. The first reference fed-batch cultivation represented the conventional fermentation process in a Brazilian Bioethanol Plant (BBP), which is carried out without aeration. The second reference cultivation was conducted under strictly anaerobic conditions (SAC). The same experimental conditions employed in FMC fermentation, including FMC’s fresh medium feeding profile, were reproduced for both BBP and SAC. Thus, after BBP and SAC experiments were carried out, the results from the three cultivations strategies can be compared to further evaluate the performance of the micro-aeration control based on the metabolic fluxes.

The experimental data showing the changes in the concentrations of glucose, biomass, ethanol, and glycerol obtained in the FMC, BBP and SAC experiments (available in Additional file 1: Figs. S3–S5 respectively) were used to obtain the performance indexes for all the experiments (Table 1) and estimate all the experimental metabolic fluxes (Box 1).

**Table 1:** Performance indexes for controlled fermentation FMC and for the reference fermentation strategies (BBP and SAC)

Performance indexes	FMC	BBP	SAC
Ethanol (g L^−1^)	87 ± 2	62.1 ± 0.3	57.2 ± 0.8
Glycerol (g L^−1^)	8.76 ± 0.01	9.33 ± 0.02	9.3 ± 0.1
Y_x/s_ (g_cells_ g_substrate_^−1^)	0.11 ± 0.001	0.08 ± 0.01	0.08 ± 0.01
Y_EtOH/s_ (g_ethanol_ g_substrate_^−1^)	0.46 ± 0.01	0.38 ± 0.03	0.39 ± 0.02
Pr_x_ (g L^−1^ h^−1^)	1.2 ± 0.2	0.59 ± 0.01	0.77 ± 0.01
Pr_p,Ethanol_ (g L^−1^ h^−1^)	7.0 ± 0.2	3.75 ± 0.02	4.05 ± 0.02
S_Index_	9.9 ± 0.8	6.6 ± 0.1	6.2 ± 0.7
Total glucose mass fed (g)	616 ± 4	552 ± 2	544 ± 2
Conversion (%)	99.6 ± 0.4	97.88 ± 0.02	99.00 ± 0.02

Ethanol and glycerol concentrations at the end of the cultivation. Overall cultivation parameters estimated: *Y*_*x/s*_ biomass yield,* Y*_*EtOH/S*_ ethanol yield,* Pr*_*X*_ volumetric cell productivity,* Pr*_*p,Ethanol*_ volumetric ethanol productivity,* S*_*Index*_ selectivity index, *S*_*Total*_ total glucose mass fed

In all fermentations, the glucose supplied was totally consumed (conversion higher than 95%) and converted mainly to ethanol, with overall yields ranging from 0.46 to 0.40 g_ethanol_ g_substrate_^−1^, whereas overall biomass yields around 0.1 g_biomass_ g_substrate_^−1^ were observed (Table 1). However, SAC and BBP lasted longer than FMC (Additional file 1: Figures S4, S5), which means that micro-aeration increased productivities for both ethanol and biomass (Table 1). In addition, the ratio between ethanol and glycerol concentrations at the end of the fermentation strategies (S_Index_, Eq. 5) of FMC (9.9) is superior to the corresponding values for BBP’s and SAC’s (6.6 and 6.2, respectively), confirming that metabolic flux towards glycerol (Fig. 2 and Additional file 1: Table S1) was reduced under controlled micro-aeration.

**Fig. 5:**
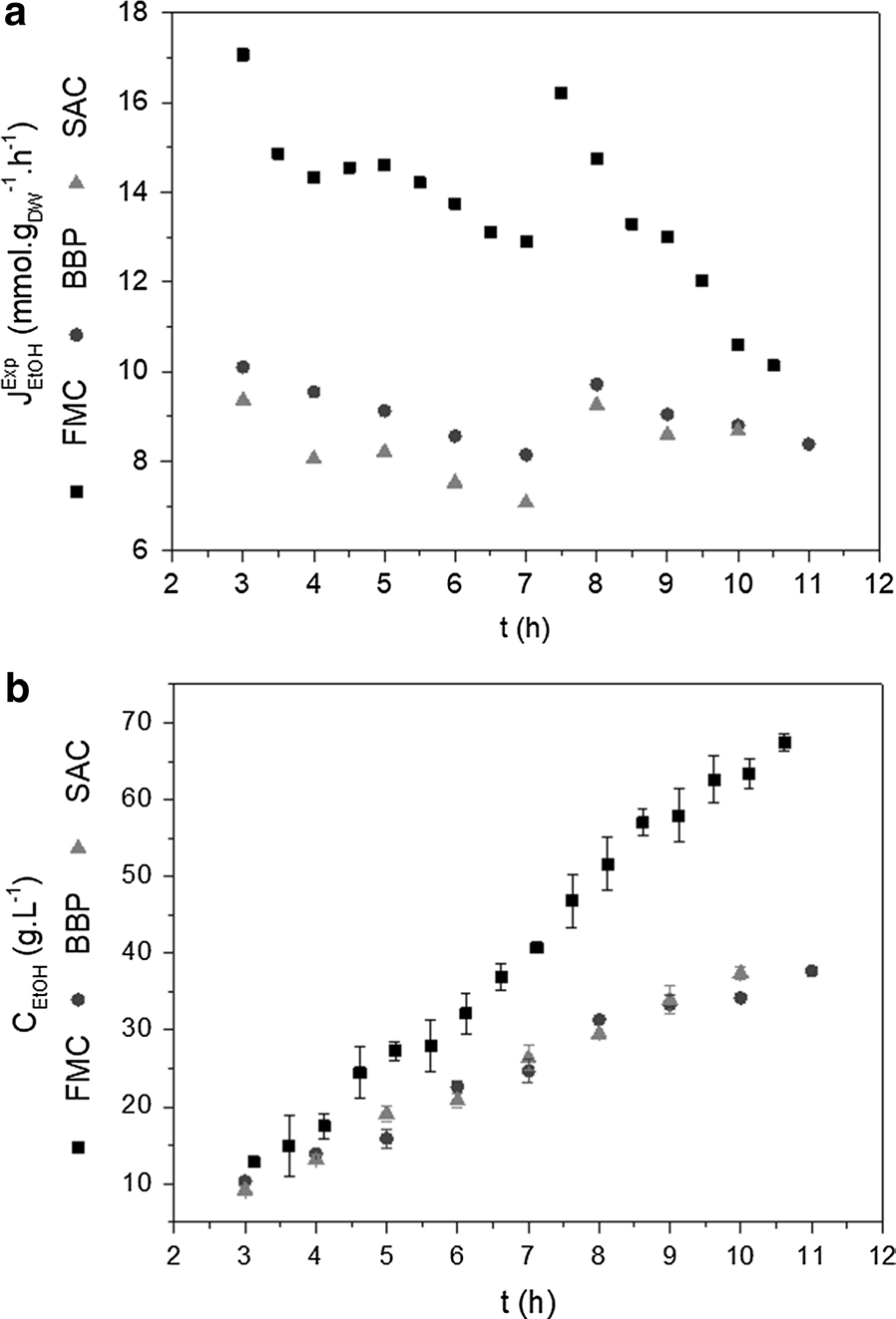
Experimental fluxes (**a**) and concentrations (**b**) of ethanol during the fed-batch phase for FMC, BBP, and SAC. **a** Experimental fluxes of ethanol $\left( {{\text{J}}_{\text{EtOH}}^{\text{Exp}} } \right)$. **b** Concentrations of ethanol $\left( {{\text{C}}{}_{\text{EtOH}}} \right)$. *FMC* flux-based micro-aeration control, *BBP “*Brazilian Bioethanol Plant”-type fermentation, *SAC* strictly anaerobic condition

Regarding metabolic fluxes, the ethanol fluxes during the feeding stage for BPP and SAC were similar and remained below 10 mmol g_DW_^−1^ h^−1^ throughout the fed-batch phase (Fig. 5a). On the other hand, for FMC, the ethanol flux remained around 14 mmol g_DW_^−1^ h^−1^ during the feeding. A better explanation for the different volumetric productivities (Table 1) can be obtained by comparing the ethanol concentrations (Fig. 5b). For FMC, the ethanol concentration increased steadily during the feeding phase, surpassing the dilution effect. Consequently, FMC ethanol productivity was 1.5-fold the values observed for BPP and SAC (Table 1). These results emphasize the potential benefits of a flux-based cultivation strategy. Indeed, high ethanol concentration and productivity were achieved in FMC due to the high ethanol flux policy successfully applied to this cultivation, as will be further discussed in the next section.

## Discussion

The modulation of oxygen fluxes following the pattern defined by the data obtained in silico using *Saccharomyces cerevisiae* iND750 GSM was effective in directing the yeast metabolism towards ethanol formation. Furthermore, the values of experimental and simulated ethanol fluxes differed by less than 15% on average (Fig. 4a), validating the initial assumption of applying the GSM steady-state simulated fluxes as a pseudo-stationary response. The proposed control strategy was successful because, in micro-aerated condition, the fluxes for biomass formation, substrate uptake, and ethanol production were limited by the oxygen supply, which, in turn, was defined by the control action.

Regarding ethanol production, the highest experimental ethanol flux achieved was 17 mmol g_DW_^−1^ h^−1^ for FMC (Fig. 5a), applying ${\text{J}}_{\text{S}}^{\text{CA}}$ of 9 mmol g_DW_^−1^ h^−1^ and ${\text{J}}_{{{\text{O}}_{2} }}^{\text{CA}}$ of 1.5 mmol g_DW_^−1^ h^−1^, which agrees with model predictions (Fig. 1a). However, this ${\text{J}}_{\text{EtOH}}^{\text{EXP}}$ value is almost half of the highest reported ethanol fluxes (up to 32 mmol g_DW_^−1^ h^−1^) in chemostat cultures under anaerobic/micro-aerated conditions using *S. cerevisiae* CBS 8066 [35]. This fast-growing *S. cerevisiae* lab strain (maximum specific growth rate of 0.45 h^−1^ at 30 °C) shows high glucose uptake fluxes (up 20 mmol g_DW_^−1^ h^−1^) under anaerobic conditions [38]. Thus, the use of high ethanol producing *S. cerevisiae* strains would certainly lead to larger experimental ethanol fluxes.

In this case, the control action proved to be sufficiently robust in managing with the limited capability of the commercial baker’s yeast used for ethanol production. Higher inlet fluxes of oxygen led to increased biomass fluxes, resulting in an RQ decrease, which was satisfactorily corrected by the implemented control loop (Fig. 1b and Box 3).

Concerning substrate feeding, glucose accumulation was observed in FMC, SAC and BBP cultures. As mentioned before, the accumulation was caused by the overestimation of the ${\text{J}}_{\text{S}}^{\text{MC}}$ by Eq. 1 (Fig. 3c) due to the influence of overestimated ${\text{J}}_{\text{X}}^{\text{MM}}$ (Fig. 4a) used for the calculation of ${\text{J}}_{{{\text{O}}_{2} }}^{\text{MC}}$ in Eq. 2. Glucose accumulation (Additional file 1: Figure S3) was intensified for ethanol concentrations over 40 g L^−1^, most certainly due to product inhibition and viability loss. This increase in glucose concentration was a consequence of misleading biomass information provided by the at-line optical density measurements, which reflected the concentrations of viable and non-viable cells. High ethanol concentrations can trigger viability loss, but the OD_600_ readings (updated at-line; Box 2) used as inputs led to overestimated C_x_ values and, consequently, to exaggerated feeding rates. Other effects of ethanol inhibition were also observed, including increased dissolved oxygen concentrations (Additional file 1: Figure S3) as well as reduced experimental substrate and biomass fluxes (Fig. 4a–c). However, no increase in glycerol metabolic flux was observed (data not shown).

Even at low concentrations (5% (v/v)), ethanol can act as an inhibitor of yeast growth, while high ethanol concentrations (10% (v/v)) greatly reduce cell viability [39]. One of the main effects of the exposure to this metabolite is an increased membrane fluidity and consequently a decrease in membrane integrity [40].

In addition, other assumptions, such as the pseudo-stationary state for substrate balance and the constant value for biomass yield, could also influence the feed flow rate calculated by the expression F (Box 2). Nevertheless, although undesirable, glucose accumulation did not hamper ethanol productivity or yield. The reason is that the cultivations were continued after the feeding of fresh medium was ended until the residual glucose was completely assimilated by the cells. Substrate accumulation is a common problem observed for different control approaches involving supplementary medium feeding in fed-batch cultures [41, 42]. The robustness of the proposed control system could be improved by on-line viable cell quantification (using a biomass sensor) or its inference using a softsensor, which should reduce the glucose accumulation.

Table 2 summarizes the main results of this work and other reported studies employing micro-aeration strategies.

**Table 2:** Main performance indexes for micro-aeration cultures carried out with *S. cerevisiae* using different substrates and medium compositions

Source	Yp/s (g_ethanol_ g_substrate_^−1^)	Pr_P, EtOH_ (g_ethanol_ L^−1^ h^−1^)	Final C_EtOH_ (g L^−1^)	Medium	Carbon source	Cultivation conditions	Yeast strain (*S. cerevisiae*)
This work (FMC)	0.46	7.0	87.2	Defined	Glucose	Fed-batch, micro-aerated	Itaiquara baker’s yeast
This work (BBP)	0.38	3.8	62.1	Defined	Glucose	Fed-batch, no gas supplied	Itaiquara baker’s yeast
This work (SAC)	0.39	4.1	57.2	Defined	Glucose	Fed-batch, strictly anaerobic	Itaiquara baker’s yeast
Ben Chaabane et al. [23]	0.44	41	65.0	Defined	Glucose	Two-stage continuous with cell recirculation, second stage micro-aerated	CBS 8066
Brandberg et al. [43]	0.47	~0.2	18.5	Defined	Glucose	Continuous with cell recirculation, micro-aerated	ATCC 96581
López-Abelairas et al. [10]	0.38	3.3	46.9	Complex	Maltose	Continuous, periodical micro-aerated	Commercial Fermentis yeast
Joannis et al. [44]	0.47	2.3	118.7	Complex	Sucrose	Very high gravity fed-batch, constant aeration (1 vvh)	C10
Deesuth et al. [45]	0.49	2.7	127.9	Complex	Sucrose	Very high gravity batch, constant aeration (0.31 vvm)	NP01

All the cited experiments were carried at 30 °C, with exception of López-Abelairas et al. [10], which was at 32.5 °C

The cultivations performed according to the metabolic flux-based control logic showed higher productivity (7.0 g_ethanol_ L^−1^ h^−1^, respectively) than those obtained in any other reported study, while the product yield (0.46 g_ethanol_ g_substrate_^−1^) was similar to the values achieved in four [23, 43–45] out of the five previously reported studies. To help contextualize these results it should be again noted that the present study used a minimum medium and a wild yeast strain.

Overall, the integration of on-line/at-line data acquisition with the trajectory predicted for the metabolic fluxes from GSM simulations within the control logic provided suitable oxygen fluxes for running a micro-aerated fermentation. The structure of the proposed control system is more flexible and comprehensive than micro-aeration tuning schemes based just on RQ measurements. The control algorithm and the mathematical model can be easily modified to handle different inputs (J_X_, J_S_, ${\text{J}}_{{\text{CO}}_2}$, and others, besides RQ), according to their availability or better matching of the metabolic response of each organism under oxygen-limited growth, both for those exhibiting homofermentative pathways (such as *S. cerevisiae*) and others following heterofermentative patterns.

## Conclusions

The proposed control system based on fluxes estimated from a genome-scale metabolic model (GSM) for *S. cerevisiae* was successfully implemented. A new supervisory system (SuperSys_Ferm) was developed and was evaluated under real fermentation conditions, with biomass concentrations ranging from 4 to 16 g_DW_ L^−1^, ethanol concentrations over 70 g L^−1^, and fed-batch/batch bioreactor operation mode. The efficacy of the control strategy was confirmed by the high ethanol yield (Y_P/S,FMC_ = 0.46 g_ethanol_ g_substrate_^−1^) and productivity (Pr_EtOH,FMC_ = 7.0 g L^−1^ h^−1^) that were achieved relative to controls.

This work has applied GSM simulations for controlling a bioprocess, manipulating both feeding and inlet gas flow rates to meet cell requirements for the desired product. As already mentioned, tuning of the control action and estimation of experimental fluxes by the supervisory system would greatly benefit from the use of an in-line biomass sensor able to determine the actual active cell concentration, which was not employed in this study. The proposed control approach is rather general and can be adapted to control several bioprocesses that require restricted aeration.

## Materials and methods

### Microorganism and cultivation media

Fed-batch cultures were carried out using fresh commercial *Saccharomyces cerevisiae* (baker’s yeast, Itaiquara brand) and minimal medium (5.0 g L^−1^ KH_2_PO_4_, 2.0 g L^−1^ MgSO_4_ 7H_2_O, 1.5 g L^−1^ urea, for both batch and feeding stages), with glucose as carbon source (30 g L^−1^ for batch cultivations and 300 g L^−1^ for the feeding medium) [46].

### Cultivation strategies

Three cultivation strategies were evaluated: (i) Metabolic Flux oriented based micro-aeration Control (FMC); (ii) “Brazilian Bioethanol Plant” type fermentation (BBP); (iii) Strictly Anaerobic Condition (SAC).

The BBP strategy reproduced the current combined fed-batch/batch cultivations used for ethanol production in most of the Brazilian sugarcane mills. Briefly, in the usual Brazilian bioethanol production process, also known as Melle-Boinot [47] a stream of concentrated yeast suspension (to achieve cell densities of 10–15% w/v) and a stream of molasse are supplied until the vessel is filled (fed-batch stage). After filling is completed, the process continues as a batch fermentation until sugar exhaustion (batch stage). During both stages no inlet gas stream is supplied [47–49]. For SAC, ultrapure nitrogen was continuously flushed into the bioreactor to ensure “true” anaerobic conditions. The description of FMC strategy is given in Methods Control strategy for FMC Cultivation. One replication of each cultivation strategy was carried out and the experiments are reproducible and representative within an expected variance for an operation of bioreactors for glycerol, ethanol and biomass concentrations (less than 15% deviation, on average, for ethanol concentrations, and less than 10% for glycerol and biomass).

### Bioreactor operation, instrumentation, and automation

The experiments were conducted in a 5-L stirred tank bioreactor (fabricated in-house) with a working volume of 3.5 L. The pH was automatically controlled (on/off, in-line GLI PRO pH meter) at 4.5 by the addition of NH_4_OH (7%) and H_3_PO_4_ (20%). Ammonium solution added during all cultures, doubling as a nitrogen source [50, 51]. The temperature was set at 30 °C. The dissolved oxygen concentration (DOC) was measured in-line using a Mettler Toledo Inpro 6800 probe connected to a CE O2 4050 transmitter. The exhaust gas composition was measured on-line using a Sick/Maihak S.710 system for CO_2_ and an additional Mettler Toledo Inpro 6800 probe for O_2_. The feed supply was provided by an Ismatec BVP pump. In all the experiments, data acquisition and monitoring/control of the instruments were achieved using SuperSys_Ferm, an in-house computational tool for flux-based control of micro-aerated fermentation processes. SuperSys_Ferm was implemented in LabView, via a Compact FieldPoint (Model cFP-2020, National Instruments), and was based on SuperSys_HCDC, which was developed to assist fed-batch bioreactor operation during high cell density cultivations [52, 53].

For all experiments, 41 g (wet mass, ~ 70% humidity) of fresh commercial baker’s yeast was suspended in 0.6 L of defined medium. This inoculum was transferred to the bioreactor leading to an initial biomass concentration of about 3.5 g L^−1^. After inoculation, the cultivations were split into three stages, as described in the following.

The first stage was a batch phase used for activation of the yeast cells and increase of the biomass concentration up to the desired range (8–12 g_DW_ L^−1^). In this stage, the oxygen saturation was kept at 30% by means of a PID controller that adjusted the stirrer speed between 200 and 1000 rpm. The air was supplied to the bioreactor at flow rates ranging from 0.5 to 5 L min^−1^, adjusted using a mass flow controller (Model GFC, Aalborg). For all experiments, as soon as the biomass concentration reached the desired range, air supply was shut down and industrial nitrogen started to be flushed to the bioreactor to reduce DOC.

For all experiments, the second and third stages mimic the current combined fed-batch/batch cultivations used for ethanol production in Brazil. The second stage started after the DOC was below 3% and was conducted in fed-batch mode. For FMC, the flow rate of the feeding medium was defined by the control strategy (further detailed in Methods Control Algorithm Design). For both BBP and SAC cultivations, the feeding profiles reproduced the one automatically implemented in the FMC culture by the control action. Since all experiments were performed using the same feeding pattern and the same overall mass of supplied substrate, this approach enabled a direct comparison of the three competing strategies. Concerning the gas supply in this cultivation stage, for FMC the inlet gas stream was a mixture of air and industrial nitrogen (99.99% N_2_ (v/v)), supplied by two mass flow controllers at the rates automatically defined by the FMC control strategy. For BBP, no gas stream was supplied, whereas for SAC ultrapure nitrogen (99.999% N_2_ (v/v)) was provided at a constant flow rate of 1 L min^−1^.

The third stage was a batch culture that followed the fed-batch phase when the feeding supply was stopped. The third phase ended when the glucose accumulated during the feeding phase, for all experiments, was totally consumed. The same strategy of gas supply already described for the second stage of FMC, BBP and SAC continued during the third stage. The stirring speed was kept constant at 400 rpm in all the experiments throughout the second and third stages.

### Concentrations of biomass, glucose and metabolites

During the experiments, samples were withdrawn and biomass formation was measured by at-line optical density readings (OD_600_) at λ = 600 nm and dry cell weight method [54]. A correlation (Eq. 3) was then used to convert OD_600_ measurements into cell mass concentration, C_X_ (g_DW_ L^−1^), to be used as input to the control action (better detailed in Methods Control Algorithm Design).

3 $${\text{C}}_{\text{x}} = \left( {0.447 \pm 0.007} \right) * {\text{OD}}_{600}       ( {\text{R}}^{2} = 0.99)$$

The concentrations of ethanol, glycerol, and glucose were measured off-line by HPLC with refractive index detection (Model 410, Waters) [55], using the following conditions: Aminex HPX-87H column (Bio-Rad); 5 mM sulfuric acid solution at a flow rate of 0.6 mL min^−1^ as the mobile phase; temperature of 50 °C.

### Experimental flux and cultivation parameters calculation

Determination of the experimental flux (${\text{J}}_{\text{i}}^{\text{Exp}}$) for each *i* component was based on Eq. 4 [56], where r_i_ is the volumetric production or consumption rate (obtained by material balances for each *i* component), in mmol of “i” L^−1^ h^−1^, and C_x_ is the biomass concentration, in g_DW_ L^−1^.

4 $${\text{J}}_{\text{i}}^{\text{Exp}} = \frac{{{\text{r}}_{\text{i}} }}{{{\text{C}}_{\text{x}} }}$$

Box 1 shows the equations used off-line to estimate ${\text{J}}_{\text{i}}^{\text{Exp}}$. To obtain the derivative terms presented for each component in Box 1, the polynomial approximation was fit to the experimental mass data as a function of time, which was further derived (Box 1, Eqs.B1.2 to B1.5). It is worth to point out that the components mass data were calculated based on the HPLC concentration results and volume correction. Besides the experimental fluxes calculated from off-line data given at Box 1, fluxes of oxygen uptake and carbon dioxide evolution were also calculated from the available on-line gas data. Experimental values of ${\text{J}}_{{\text{O}}_2}$ and ${\text{J}}_{{\text{CO}}_2}$ are essential for running the control algorithm and their estimation is given at equations B2.4 and B2.5 (Box 2).

An additional index, denominated Overall Selectivity Index (S_Index_) was defined as the ratio between the concentrations of ethanol and glycerol at the end of the cultivation, as stated in Eq. 5, and represented a measure of the metabolic shift towards glycerol formation under the different policies of oxygen supply applied.

5 $${\text{S}}_{\text{Index}}^{{}} = \frac{{{\text{C}}_{{{\text{EtOH}}, {\text{Final}}}} }}{{{\text{C}}_{{{\text{Gly}},{\text{Final}}}} }}$$

The products yields (Y_i/s_) (Eq. 6) and volumetric productivities, Pr_p,i_ (Eq. 7) were obtained also as overall indexes, according to the definitions described below.

6 $${\text{Y}}_{{{\text{i}}/{\text{S}}}} = \frac{{\left( {{\text{Final mass}} - {\text{Initial mass}}} \right)_{{{\text{Product}},{\text{i}}}} }}{{({\text{Total mass fed}} - {\text{Residual mass}})_{\text{Glucose}} }}$$

7 $${ \Pr }_{{{\text{p}},{\text{i}}}} = \frac{{{\text{Final concentration}}_{{{\text{Product}},{\text{i}}}} }}{\text{Total cultivation time}}$$

### Control strategy for FMC cultivation

Besides experimental fluxes (${\text{J}}_{\text{i}}^{\text{Exp}}$), three different kinds of fluxes are presented in this paper They were generated using data from: (1) Metabolic Model simulations (${\text{J}}_{\text{i}}^{\text{MM}}$); (2) On-line Estimation using the supervisory software (${\text{J}}_{\text{i}}^{\text{CA}}$); and (3) Mathematical Correlations (${\text{J}}_{\text{i}}^{\text{MC}}$); these fluxes are used as inputs for the control algorithm to manipulate the flow rates of air (Q_AIR_), nitrogen (Q_N2_), and fresh medium feed (F). The nomenclature included at the beginning of the article provides a detailed description of all fluxes used in this work and their symbols.

${\text{J}}_{\text{i}}^{\text{MM}}$ are represented in Fig. 1a, and they were estimated off-line from metabolic model simulations (detailed in the next section). ${\text{J}}_{\text{i}}^{\text{MC}}$ were the fluxes calculated on-line by mathematical correlations. These correlations were generated off-line from selected ${\text{J}}_{\text{i}}^{\text{MM}}$ (Fig. 1a). ${\text{J}}_{\text{i}}^{\text{CA}}$ were the fluxes calculated on-line by the control algorithm, which was set up to drive the control action (Fig. 1b, Boxes 2 and 3).

### Metabolic model simulations

The control strategy implemented in the FMC experiment was based on the *S. cerevisiae* cell metabolic responses. An overview of the proposed control and experimental approach is shown in Fig. 1. Figure 1a presents the in silico studies of *S. cerevisiae* metabolism performed off-line with glucose as carbon source, using Optflux 3.2.7 software [34]. The genome-scale metabolic model (GSM) of *S. cerevisiae* iND750 [33] was used, together with the parsimonious flux balance analysis optimization method (pFBA) [57]. The objective function that maximizes the biomass flux was chosen, assuming that it may provide a better emulation of the actual behavior of a yeast cell struggling to grow in the environment of the bioreactor. Another meaningful objective function would be the maximization of the ethanol flux, but within our concept, the actuation of the advanced control system would be responsible for the task of maximizing ethanol yields.

The simulations focused on mapping the metabolism, given inlet fluxes of oxygen and glucose ranging from 15 (fully aerobic) to 0.001 (severely oxygen-limited) mmol O_2_ g_DW_^−1^ h^−1^, and from 1 to 50 mmol glucose g_DW_^−1^ h^−1^, respectively [35]. Several simulations were carried out and the simulated responses of metabolic ethanol flux (${\text{J}}_{\text{EtOH}}^{\text{MM}}$) and respiratory quotient (RQ^MM^) were collected for each ${\text{J}}_{\text{S}}^{\text{MM}}$ and ${\text{J}}_{{{\text{O}}_{2} }}^{\text{MM}}$ used as the environmental conditions. ${\text{J}}_{\text{S}}^{\text{MM}}$ was kept constant at fixed values and ${\text{J}}_{{{\text{O}}_{2} }}^{\text{MM}}$ was gradually reduced until a maximum ${\text{J}}_{\text{EtOH}}^{\text{MM}}$ was observed. This procedure was repeated for several ${\text{J}}_{\text{S}}^{\text{MM}}$ (Step 1, illustrated in Table SM1 available in Additional file 1). The optimal inlet fluxes ${\text{J}}_{{{\text{O}}_{2} }}^{\text{MM}}$ for each ${\text{J}}_{\text{S}}^{\text{MM}}$ were identified considering the range from 85 to 100% of the maximum ${\text{J}}_{\text{EtOH}}^{\text{MM}}$ obtained in the simulations (Step 2, illustrated in Additional file 1: Table S2).

In Fig. 1a, it is possible to observe a gray shaded area, which was generated by compiling all simulation results for several ${\text{J}}_{\text{S}}^{\text{MM}}$ and the corresponding optimal ${\text{J}}_{{{\text{O}}_{2} }}^{\text{MM}}$. The RQ^MM^, ${\text{J}}_{{{\text{O}}_{2} }}^{\text{MM}}$, and ${\text{J}}_{\text{S}}^{\text{MM}}$ data into the 85% to 100% boundary were further used to obtain the mathematical correlations presented in the results, which were used to support the control action (Fig. 1b).

### Control algorithm design

The flux-based micro-aerated control fermentation (FMC) was implemented as an on-line closed-loop type control policy [58], with RQ as the controlled variable (${\text{RQ}}_{{}}^{\text{CA}}$) (Fig. 1b), having the O_2_ and CO_2_ molar fractions in the exhaust gas as input variables (Box 2). The link between metabolic model simulations and the control action can be seen in Eqs. B2.1 and B2.3, where ${\text{J}}_{{{\text{O}}_{2} }}^{\text{MC}}$ (on-line estimated by the RQ-based correlation given at Eq. 2) and ${\text{J}}_{\text{S}}^{\text{MC}}$ (on-line estimated by Eq. 1) were input variables used to update air and feeding medium flow rates, respectively. In addition, the control actuated to keep RQ at the desired range. The entire supervisory system was assembled in SuperSys_Ferm.

The algorithm which was set up to run the on-line flux-based control for the FMC experiment is outlined in Box 2. The rO_2_ equation was based on the combination of the metabolic flux definition and mass balance (Box 2, Eq. B2.2). Thus, the Q_AIR_ was manipulated according to Eq. B2.3 and the Q_N2_ increased automatically (observing the CO_2_ molar fraction upper detection limit provided by the gas analyzer) during the experiment’s second and third stages (as described in Methods Cultivation Strategies). The respiratory quotient (Box 2, Eq. B2.6) was calculated on-line as the ratio between carbon dioxide evolution rate (CER) (${\text{r}}_{{{\text{CO}}_{2} }}$) and the oxygen uptake rate (OUR) (${\text{r}}_{{{\text{O}}_{2} }}$) or, similarly, ${\text{J}}_{{{\text{CO}}_{2} }}^{\text{CA}}$ and ${\text{J}}_{{{\text{O}}_{2} }}^{\text{CA}}$. Both ${\text{J}}_{{{\text{O}}_{2} }}^{\text{CA}}$ and ${\text{J}}_{{{\text{CO}}_{2} }}^{\text{CA}}$ were obtained on-line by a pseudo steady-state material balance for the continuous gas phase (Box 2, B2.4 and B2.5, respectively). The flow rate of fresh medium (F) as a function of ${\text{J}}_{\text{S}}^{\text{MC}}$ was calculated using Eq. B2.1 (Box 2). The F equation is the solution of a second-order polynomial, which was obtained from a pseudo steady-state material balance for the substrate, together with the cell yield definition.

Cell masses m_X_ and m_X,0_ (Box 2, Eq. B1.1) were obtained at-line using OD measurements. The biomass yield (Y_X/S_) and the volume (V) were estimated at-line by the supervisory system. The value of C_SF_ was set to the actual glucose concentration in the feeding medium (~ 300 g L^−1^) measured before the experiment was started.

### Control adjustments and heuristics

Several adjustments were made to provide better performance for the control system. The first modification was the insertion of an 80-point moving average filter [59] on the input variables: inlet pressure (P_in_), outlet temperature (T_out_), outlet oxygen fraction (${\text{y}}_{{{\text{O}}_{ 2} , {\text{ out}}}}$), outlet carbon dioxide fraction (${\text{y}}_{{{\text{CO}}_{ 2} , {\text{ out}}}}$), inlet oxygen fraction (${\text{y}}_{{{\text{O}}_{ 2} , {\text{ in}}}}$), and inlet carbon dioxide fraction (${\text{y}}_{{{\text{CO}}_{ 2} , {\text{ in}}}}$). This was done in order to maintain system stability and protect the equipment (e.g.: mass flow controllers, peristaltic pump) against sudden changes or oscillations [26]. The second adjustment was the addition of a control delay with Δt_lag_ = 60 s. This value refers to the time interval required for a given change in the gas stream composition to travel through the entire piping system and to be detected by the gas analyzer.

The pseudo-code of the RQ control implemented in the FMC cultivation is shown in Box 3. It can be separated into three conditions, as follows.

In condition (1), the heuristics first determine if the on-line calculated RQ (${\text{RQ}}_{{}}^{\text{CA}} )$ is between the established boundaries. If this statement is true, ${\text{J}}_{\text{S}}^{\text{MC}}$ and ${\text{J}}_{{{\text{O}}_2}}^{\text{MC}}$ are updated along with F and Q_AIR_ (Box 2, Eqs. B2.1 and B2.3, respectively). If the statement in (1) is false, then conditions (2) and (3) are examined.

Condition (2) is true when ${\text{RQ}}_{{}}^{\text{CA}}$ is lower than the lower boundary, with the heuristics then calculating ${\text{J}}_{{{\text{CO}}_2}}^{\text{CA}}$ at an instant (n) (${\text{J}}_{{{\text{CO}}_{2} }}^{\text{CA}}$ _new) and comparing it to ${\text{J}}_{{{\text{CO}}_{2} }}^{\text{CA}}$ at an instant (n-1) (${\text{J}}_{{{\text{CO}}_{2} }}^{\text{CA}}$ _previous). If $\left( {{\text{J}}_{{{\text{CO}}_{2} }}^{\text{CA}} } \right)$_n_ < $\left( {{\text{J}}_{{{\text{CO}}_{2} }}^{\text{CA}} } \right)$_n−1_ is true, the last ${\text{J}}_{\text{S}}^{\text{MC}}$, ${\text{J}}_{{{\text{O}}_{2} }}^{\text{MC}}$, Q_AIR_, and F are maintained. If false, it means that respiration might be under increasing activation. So, ${\text{J}}_{{{\text{O}}_{2} }}^{\text{MC}}$ is decremented by 0.5 mmol g_MS_^−1^ h^−1^ to increase RQ at the next iteration, and ${\text{J}}_{\text{S}}^{\text{MC}}$, Q_AIR_, and F are updated. The ${\text{RQ}}_{{}}^{\text{CA}}$’s lower boundary is based on the 90% region of the maximum ${\text{J}}_{\text{EtOH}}^{\text{MM}}$ (explained in Methods Metabolic Models Simulations).

Condition (3) is true when ${\text{RQ}}_{{}}^{\text{CA}}$ is higher than the upper boundary. In this case, ${\text{J}}_{{{\text{O}}_2}}^{\text{MC}}$ undergoes an increment of 0.5 mmol g_MS_^−1^ h^−1^, to decrease RQ at the next iteration, and ${\text{J}}_{\text{S}}^{\text{MC}}$, Q_AIR_, and F are also updated. The ${\text{RQ}}_{{}}^{\text{CA}}$’s upper boundary was established due to the mass flow meters operational restrictions.

## Supplementary information

**Additional file 1: Figure S1.** Linear correlation used to estimate ${\text{J}}_{\text{S}}^{\text{MC}}$ from ${\text{J}}_{{{\text{O}}_{2} }}^{\text{MC}}$ input data. Each state in Fig SM1 corresponds to a different solution of the GSM, which provides different fluxes of ethanol and biomass. The multiplicity of metabolic states in this figure reflects the use of an 85–100% range of ${\text{J}}_{\text{EtOH}}^{\text{MM}}$. **Figure S2.** Hyperbolic correlation employed to estimate ${\text{J}}_{{{\text{O}}_{2} }}^{\text{MC}}$ from RQ^CA^ (control loop action). The influence of RQ^CA^ on ${\text{J}}_{\text{EtOH}}^{\text{MM}}$ is also shown. **Figure S3.** FMC cultivation profile: biomass (black square), ethanol (red circle), glycerol (brown inverted triangle), glucose (blue triangle), dissolved oxygen (green line). (a) Beginning of control action; (b) end of fresh medium feeding. **Figure S4.** BBP cultivation profile: biomass (black square), ethanol (red circle), glycerol (brown inverted triangle), glucose (blue triangle), dissolved oxygen (green line). (a) Beginning of fresh medium feeding (F profile reproduced from FMC); (b) end of fresh medium feeding. **Figure S5.** SAC cultivation profile: biomass (black square), ethanol (red circle), glycerol (brown inverted triangle), glucose (blue triangle), dissolved oxygen (green line). (a) Beginning of fresh medium feeding (F profile reproduced from FMC); (b) end of fresh medium feeding. **Table S1.** Results of in silico studies (Step 1) using the IND750 metabolic model (DUARTE, HERRGÂRD and PALSSON, 2004). Influence of the oxygen inlet fluxes on the biomass, CO_2_, and ethanol production fluxes (for an inlet glucose flux of 3 mmol g_DW_^−1^ h^−1^). *Conditions for the maximum ethanol flux production. **Table S2.** Ethanol fluxes for different oxygen inlet fluxes (Step 2) (considering an inlet glucose flux of 3 mmol g_DW_^−1^ h^−1^). *Inlet O_2_ flux that led to 90% of the maximum ethanol production flux observed. ^+^ Maximum ethanol flux production ${\text{J}}_{\text{EtOH}_{\text{MAX}}}^{\text{MM}}$.

## Data Availability

All data presented in this article, including Additional file 1, are also available from the corresponding author on reasonable request.

## Abbreviations

cFP: Compact Fieldpoint
C_SF_: feed flow substrate concentration (g L^−1^)
C_x_: at-line dry cell mass concentration (g_DW_ L^−1^)
DOC: In-line dissolved oxygen concentration (% saturation)
BBP: “Brazilian Bioethanol Plant”-type fermentation
EtOH: ethanol
F: fresh medium feeding flow (L h^−1^)
FMC: flux-based micro-aeration control
GSM: genome-scale metabolic model
Gly: glycerol
HPLC: high performance liquid chromatography
${\rm J}_{{{\rm O}_2}}^{\rm CA}$: control on-line estimated O_2_ flux (mmol g_DW_^−1^ h^−1^)
${\rm J}_{{{\rm CO}_2}}^{\rm CA}$: control on-line estimated CO_2_ flux (mmol g_DW_^−1^ h^−1^)
${\rm J}_{\rm EtOH}^{\rm Exp}$: off-line experimental ethanol flux (mmol g_DW_^−1^ h^−1^)
${\rm J}_{\rm X}^{\rm Exp}$: off-line experimental biomass flux (mmol g_DW_^−1^ h^−1^)
${\rm J}_{\rm Gly}^{\rm Exp}$: off-line experimental glycerol flux (mmol g_DW_^−1^ h^−1^)
${\rm J}_{\rm X}^{\rm Exp}$: off-line experimental biomass flux (mmol g_DW_^−1^ h^−1^)
${\rm J}_{{{\rm O}_{2} }}^{\rm MC}$: mathematical correlation O_2_ flux (mmol g_DW_^−1^ h^−1^)
${\rm J}_{\rm S}^{\rm MC}$: mathematical correlation substrate flux (mmol g_DW_^−1^ h^−1^)
$${\rm J}_{{{\rm O}_2}}^{\rm MC}$$ _new: $${\text{J}}_{{{\text{O}}_2}}^{\text{MC}}$$ at a moment (n)
$${\rm J}_{{{\rm O}_2}}^{\rm MC}$$ _previous: $${\text{J}}_{{{\text{O}}_2}}^{\text{MC}}$$ at a moment (n − 1)
$${\rm J}_{\rm S}^{\rm MC}$$ _new: $${\text{J}}_{\text{S}}^{\text{MC}}$$ at a moment (n)
$${\rm J}_{\rm S}^{\rm MC}$$ _previous: $${\text{J}}_{\text{S}}^{\text{MC}}$$ at a moment (n − 1)
${\rm J}_{{{\rm O}_2}}^{\rm MM}$: metabolic model O_2_ flux (mmol g_DW_^−1^ h^−1^)
${\rm J}_{{{\rm CO}_2}}^{\rm MM}$: metabolic model CO_2_ flux (mmol g_DW_^−1^ h^−1^)
${\rm J}_{\rm EtOH}^{\rm MM}$: metabolic model ethanol flux (mmol g_DW_^−1^ h^−1^)
${\rm J}_{\rm Gly}^{\rm MM}$: metabolic model glycerol flux (mmol g_DW_^−1^ h^−1^)
${\rm J}_{\rm S}^{\rm MM}$: metabolic model substrate flux (mmol g_DW_^−1^ h^−1^)
${\rm J}_{\rm X}^{\rm MM}$: metabolic model biomass flux (mmol g_DW_^−1^ h^−1^)
m_x_: off-line dry cell weight (g_DW_)
m_s_: substrate weight (g)
m_xin_: dry cell weight at the beginning of the feeding (g_DW_)
${{ \dot{\rm{n}}}}_{{{\rm O}_2,{\rm in}}}$: inlet oxygen molar flow (mol h^−1^)
${{ \dot{\rm{n}}}}_{{{\rm O}_2,{\rm out}}}$: outlet oxygen molar flow (mol h^−1^)
OD_600_: at-line Optical density at 600 nm
P_in_: on-line Inlet pressure (atm)
Pr_pEthanol_: ethanol volumetric productivity (g L^−1^ h^−1^)
P_out_: outlet pressure (atm)
Pr_X_: cell volumetric productivity (g L^−1^ h^−1^)
Q_gas in_: inlet gas flow (L min^−1^)
Q_gas out_: outlet gas flow (L min^−1^)
Q_AIR_: air flow (L min^−1^)
Q_AIR__previous: calculated air flow at a moment (n-1)
R: universal gas constant (atm L mol^−1^ K^−1^)
r_i_: component *i* production or consumption rate (mmol L^−1^ h^−1^)
${\text{r}}_{{{\text{CO}}_{ 2} }}$: carbon evolution rate (mmol L^−1^ h^−1^)
${\text{r}}_{{{\text{O}}_{ 2} }}$: oxygen uptake rate (mmol L^−1^ h^−1^)
RQ: respiratory quotient
RQ^CA^: respiratory quotient calculated using on-line data
RQ^MM^: respiratory quotient predicted by the metabolic model
RQ_LowerBound: lower RQ boundary
RQ_UpperBound: upper RQ boundary
S: substrate
SAC: strictly Anaerobic Condition
S_Index_: selectivity index
t: time (h)
T_in_: inlet temperature (K)
T_out_: in-line Outlet temperature (K)
V: control estimated Volume (L)
vvm: vessel volumes per minute
vvh: vessel volumes per hour
${\text{y}}_{{{\text{CO}}_{{ 2 { }}} {\text{in}}}}$: control estimated Inlet CO_2_ fraction
${\text{y}}_{{{\text{CO}}_{ 2} {\text{ out}}}}$: on-line Outlet CO_2_ fraction
$${\text{y}}_{{{\text{O}}_{ 2} {\text{ AIR}}}}$$: air O_2_ fraction
${\text{y}}_{{{\text{O}}_{ 2} {\text{ in}}}}$: control estimated Inlet O_2_ fraction
${\text{y}}_{{{\text{O}}_{ 2} {\text{ out}}}}$: on-line Outlet O_2_ fraction
Y_P/S_: product yield (g_P_ g substrate^−1^)
Y_X/S_: biomass yield (g_DW_ g substrate^−1^)
Δt: time interval (program iteration) (h)
Δt_lag_: time delay (s)
