# Supplementary material for: Metabolic fluxes-oriented control of bioreactors: a novel approach to tune micro-aeration and substrate feeding in fermentations
Source: Microb Cell Fact. 2019 Sep 4;18:150. doi: 10.1186/s12934-019-1198-6 (PMC6724378; doi:10.1186/s12934-019-1198-6)
Supplement: Supplementary file 1 — Additional file 1: Figure S1. Linear correlation used to estimate ${\text{J}}_{\text{S}}^{\text{MC}}$JSMC from ${\text{J}}_{{{\text{O}}_{2} }}^{\text{MC}}$JO2MC input data. Each state in Fig SM1 corresponds to a different solution of the GSM, which provides different fluxes of ethanol and biomass. The multiplicity of metabolic states in this figure reflects the use of an 85–100% range of ${\text{J}}_{\text{EtOH}}^{\text{MM}}$JEtOHMM. Figure S2. Hyperbolic correlation employed to estimate ${\text{J}}_{{{\text{O}}_{2} }}^{\text{MC}}$JO2MC from RQCA (control loop action). The influence of RQCA on ${\text{J}}_{\text{EtOH}}^{\text{MM}}$JEtOHMM is also shown. Figure S3. FMC cultivation profile: biomass (black square), ethanol (red circle), glycerol (brown inverted triangle), [file 12934_2019_1198_MOESM1_ESM.docx]

**Metabolic fluxes-oriented control of bioreactors: a novel approach to tune micro-aeration and substrate feeding in fermentations**

Thiago José Barbosa Mesquita^1^, Cíntia Regina Sargo^2^, José Roberto Fuzer Neto^1^, Sheyla Alexandra Hidalgo Paredes^1^, Roberto de Campos Giordano^1^, Antonio Carlos Luperni Horta^1^, Teresa Cristina Zangirolami^1^

^1^ Graduate Program of Chemical Engineering, Federal University of São Carlos (PPGEQ-UFSCar), Rodovia Washington Luís, km 235, 13565-905, São Carlos, SP, Brazil

^2^ Graduate Program of Chemical Engineering – Institute of Chemistry, Federal University of Goiás (PPGEQ/IQ – UFG) Avenida Esperança, Campus Samambaia, 74690-900, Goiânia, GO, Brasil

^*^Corresponding author:

E-mail: teresacz@ufscar.br

Phone: +55 16 3351 8710

Fax: +55 16 3351 8266

**Additional Files 1**

Table S1 – Results of in silico studies (Step 1) using the IND750 metabolic model (DUARTE, HERRGÂRD and PALSSON, 2004). Influence of the oxygen inlet fluxes on the biomass, CO_2_ and ethanol production fluxes (for an inlet glucose flux of 3 mmol.g_DW_^-1^.h^-1^). † Conditions for the maximum ethanol flux production.

| **Simulations** | **Inlet Fluxes**  **(mmol g_DW_^-1^ h^-1^)** | | **Results**  **(mmol g_DW_^-1^ h^-1^)** | | | | |
| --- | --- | --- | --- | --- | --- | --- | --- |
|  | Glucose  ($J_{S}^{\mathrm{MM}})$ | O_2_  $(J_{O_{2}}^{\mathrm{MM}}$) | Biomass  $(J_{X}^{\mathrm{MM}})$ | CO_2_  $(J_{CO_{2}}^{\mathrm{MM}}$) | Ethanol  $(J_{\mathrm{EtOH}}^{\mathrm{MM}})$ | Glycerol | RQ |
|  |  |  |  |  |  | ${(J}_{\mathrm{Gly}}^{\mathrm{MM}})$ |  |
| 1 | -3 | -8 | 0.28 | 8.1 | 0 | 0 | 1.01 |
| 2 | -3 | -7.3 | 0.29 | 7.51 | 0.002 | 0 | 1.03 |
| 3 | -3 | -7 | 0.28 | 7.41 | 0.21 | 0 | 1.06 |
| 4 | -3 | -3 | 0.16 | 6.14 | 3.03 | 0 | 2.05 |
| 5 | -3 | -1 | 0.1 | 5.51 | 4.44 | 0 | 5.51 |
| 6 | -3 | -0.5 | 0.084 | 5.36 | 4.80 | 0 | 10.72 |
| 7 | -3 | -0.1 | 0.068 | 5.28 | 5.11 | 0.0002 | 52.80 |
| 8 | -3 | -0.05 | 0.065 | 5.28 | 5.12 | 0.0002 | 105.60 |
| †**9** | **-3** | **-0.005** | **0.062** | **5.28** | **5.15** | **0.0002** | **1056.00** |
| 10 | -3 | -0.003 | 0.054 | 4.61 | 4.41 | 0.0002 | 1536.67 |
| 11 | -3 | -0.0005 | 0.009 | 1.88 | 0.73 | 0.0296 | 3760.00 |

Table S2 – Ethanol fluxes for different oxygen inlet fluxes (Step 2) (considering an inlet glucose flux of 3 mmol.g_DW_ ^-1^.h^-1^). † Inlet O_2_ flux that led to 90% of the maximum ethanol production flux observed. ‡ Maximum ethanol flux production $J_{EtOH MÁX}^{MM}$.

| **Simulation** | **Inlet Fluxes**  **(mmol g_DW_^-1^ h^-1^)** | | **Results**  **(mmol g_DW_^-1^ h^-1^)** | |
| --- | --- | --- | --- | --- |
|  | Glucose  ($J_{S}^{\mathrm{MM}})$ | O_2_  $(J_{O_{2}}^{\mathrm{MM}}$) | Ethanol  $(J_{\mathrm{EtOH}}^{\mathrm{MM}})$ | % EtOH  (regarding the EtOH_máx,_ $J_{EtOH MÁX}^{\mathrm{MM}}$) |
| 1 | -3 | -3 | 3.03 | 58.88 |
| 2 | -3 | -1 | 4.44 | 86.21 |
| †**3** | **-3** | **-0.7** | **4.66** | **90.49** |
| 4 | -3 | -0.5 | 4.80 | 93.20 |
| 5 | -3 | -0.1 | 5.11 | 99.22 |
| 6 | -3 | -0.05 | 5.12 | 99.42 |
| ‡**7** | **-3** | **-0.005** | **5.15** | **100.00** |

Figure S1 - Linear correlation used to estimate $J_{S}^{\mathrm{MC}}$ from $J_{O2}^{\mathrm{MC}}$ input data. Each state in Fig SM1 corresponds to a different solution of the GSM, which provides different fluxes of ethanol and biomass. The multiplicity of metabolic states in this figure reflects the use of an 85-100% range of $J_{\mathrm{EtOH}}^{\mathrm{MM}}$.

Figure S2 - Hyperbolic correlation employed to estimate $J_{O2}^{\mathrm{MC}}$ from RQ^CA^ (control loop action). Influence of RQ^CA^  on $J_{\mathrm{EtOH}}^{\mathrm{MM}}$ also showed

Figure S3 - FMC cultivation profile: biomass (■), ethanol (●), glycerol (▼), glucose (▲), dissolved oxygen (▬). (a) Beginning of control action, (b) end of fresh medium feeding.

Figure S4 - BBP cultivation profile: biomass (■), ethanol (●), glycerol (▼), glucose (▲), dissolved oxygen (▬). (a) Beginning of fresh medium feeding (F profile reproduced from FMC); (b) end of fresh medium feeding.

Figure S5 - SAC cultivation profile: biomass (■), ethanol (●), glycerol (▼), glucose (▲), dissolved oxygen (▬). (a) Beginning of fresh medium feeding (F profile reproduced from FMC); (b) end of fresh medium feeding.

**Box 2 Equations Derivations**

For Box 1, the dry cell mass equation B1.1 was obtained by linear regression using experimental data for biomass concentration (C_x_) (in dry cell weight) and the corresponding optical density values. All the other equations (B1.2 to B1.5) were obtained by individual component mass balance, combined with flux definition $\left( J_{i}=\frac{r_{i}}{C_{X}} \right)$.

Regarding the liquid phase, the substrate flux (J_S_, in mmol.g_DW_^-1^.h^-1^) is used to estimate the volumetric substrate consumption rate (r_S_, in mmol.L^-1^.h^-1^), defined at Eq. I.

$\text{r}_{\text{S}}\text{=}\text{J}_{\text{S}}\text{C}_{\text{X}}$ (I)

During the feeding stage, the r_S_ can be estimated by the substrate material balance (Cs, in g.L^-1^), resulting in Eq. (II). Assuming the hypothesis of the pseudo-steady state for Cs, we have:

$$\frac{\text{d(Cs.V)}}{\text{dt}}\text{=F}\text{C}_{\text{SF}}\text{-}\text{ }\text{r}_{\text{s}}\text{V-}\text{F}_{\text{w}}\text{Cs}$$

$$\text{V}\frac{\text{d(Cs)}}{\text{dt}}\text{+Cs}\frac{\text{d(V)}}{\text{dt}}\text{=F}\text{C}_{\text{SF}}\text{- }\text{r}_{\text{s}}\text{V}\text{-F}_{w}\text{Cs}$$

$$\text{V}\frac{\text{d(Cs)}}{\text{dt}}\text{+Cs(F-}F_{w}\text{)=F}\text{C}_{\text{SF}}\text{- }\text{r}_{\text{s}}\text{V}\text{-F}_{w}\text{Cs}$$

$$\text{V}\frac{\text{d(Cs)}}{\text{dt}}\text{=F(}\text{C}_{\text{SF}}\text{-Cs)- }\text{r}_{\text{s}}\text{V}$$

$$\text{F}\left( \text{C}_{\text{SF}}\text{-Cs} \right)\text{- }\text{r}_{\text{s}}\text{V=0}$$

$\text{r}_{\text{S}}\text{=Js.Cx=}\frac{\text{F}\left( \text{C}_{\text{SF}}\text{-}\text{C}_{\text{S}} \right)}{\text{V}}$ (II)

where F is the volumetric flow rate of feed medium (L.h^-1^), C_SF_ is the substrate concentration in the feeding (g.L^-1^) and F_w_ is the sample withdrawal rate.

The substrate concentration can be related to the biomass yield coefficient (Y_X/S_), as shown in Eq. (III).

$\text{Y}_{\text{X/S}}\text{=}\frac{\text{m}_{\text{X}}\text{-}\text{m}_{\text{X,in}}}{\text{m}_{\text{SF}}\text{-}\text{C}_{\text{s}}\text{V}}$ (III)

where m_X_ is the mass of cells in suspension during the feeding phase (g); m_Xin_ is the mass of cells present at the beginning of feed (g) and m_SF_ is the mass of substrate supplied during feeding, which can be estimated by:

$\text{m}_{\text{SF}}\text{=}\text{C}_{\text{SF}}\int\text{F*dt}$ (IV)

F (feeding rate, L.h^-1^) can be taken as constant, if it is updated in fixed, short time intervals (dt $\cong$ Δt_F_). So, if at each new time interval, a new feeding phase started, Eqs. III and IV can be combined. A second order polynomial is obtained, and its algebraic negative solution turns F a function of the substrate metabolic flux (Eq. V or Eq. B2.1, Box 2).

$$\text{Y}_{\text{X/S}}\text{(}\text{C}_{\text{SF}}\text{.F.}\text{∆t}_{\text{F}}\text{-}\text{C}_{\text{s}}\text{V)=}\text{m}_{\text{X}}\text{-}\text{m}_{\text{X,in}}$$

$${\text{Y}_{\text{X/S}}\text{.C}}_{\text{s}}\text{.V=}\text{Y}_{\text{X/S}}\text{.}\text{C}_{\text{SF}}\text{.F.}\text{∆t}_{\text{F}}\text{-(}\text{m}_{\text{X}}\text{-}\text{m}_{\text{X,in}}\text{)}$$

$$\text{C}_{\text{SF}}\text{-}\frac{\text{J}_{\text{S}}\text{.Cx.V}}{\text{F}}\text{=C}_{\text{S}}$$

$$\text{(}\text{C}_{\text{SF}}\text{-}\frac{\text{J}_{\text{S}}\text{.Cx.V}}{\text{F}}\text{)}\text{Y}_{\text{X/S}}\text{.V=}\text{Y}_{\text{X/S}}\text{.}\text{C}_{\text{SF}}\text{.F.}\text{∆t}_{\text{F}}\text{-(}\text{m}_{\text{X}}\text{-}\text{m}_{\text{X,in}}\text{)}$$

$\text{F=}\frac{\left[ \left( \frac{\text{m}_{\text{x}}\text{-}\text{m}_{\text{x,0}}}{\text{Y}_{\text{X/S}}\text{ }}\text{ }\frac{\text{1}}{\text{V}}\text{+}\text{C}_{\text{SF}} \right) \right]\text{-}\sqrt{\left( \frac{\text{m}_{\text{x}}\text{-}\text{m}_{\text{x,0}}}{\text{Y}_{\text{X/S}}}\text{ }\frac{\text{1}}{\text{V}}\text{+}\text{C}_{\text{SF}} \right)^{\text{2}}\text{-4}\left( \frac{\text{C}_{\text{SF}}\text{∆t}_{\text{F}}}{\text{V}} \right)\left( \text{C}_{\text{x}}\text{J}_{\text{S}}\text{V} \right)}}{\text{2}\left( \frac{\text{C}_{\text{SF}}\text{∆t}_{\text{F}}}{\text{V}} \right)}$ (V)

Yet, regarding the gas phase equations shown in Box 2, the calculation procedures are described as follows. Based on the values selected by the simulation for O_2_ flux (J_O2_ in mmol.g_DW_^-1^.h^-1^), it is possible to estimate the oxygen uptake rate (r_O2_, in mmol.L^-1^.h^-1^) using Eq. VI. Cell concentration is estimated by off-line optical density readings as explained in the manuscript.

$\text{r}_{\text{O2}}\text{=}\text{J}_{\text{O2}}\text{C}_{\text{X}}$ (VI)

The inlet oxygen flow (Q_O2,,in_) was calculated by the combination of the ideal gas law (Eq.VII and Eq. VIII) and molar mass balance r_O2_ (Eq. IX).

$\text{Q}_{\text{O2,in}}\text{=}\frac{\dot{n}_{\text{O2,in}}\text{*R*}\text{T}_{\text{in}}}{\text{P}_{\text{in}}}$ (VII)

${\text{Q}_{\text{O2,in}}\text{=}\text{y}_{\text{O2,N2}}\text{Q}}_{\text{N2}}\text{+}{\text{y}_{\text{O2,Air}}\text{Q}}_{\text{Air}}$ (VIII)

$\text{r}_{\text{O2}}\text{=}\frac{\dot{n}_{\text{O2,in}}\text{-}\dot{n}_{\text{O2,out}}}{\text{V}}$ (IX)

In which, T_in_ is the inlet gas stream temperature; P_in_ is the inlet pressure; R is the ideal gas constant; y_O2,N2_ is the oxygen fraction in the N_2_ gas; y_O2,Air_ is the oxygen fraction in the air; Q_N2_ is the N_2_ flow rate; Q_Air_ is the air flow rate; $\dot{n}_{\text{O2,in}}$ is the inlet O_2_ molar flow;$\dot{n}_{\text{O2,out}}$ is the outlet O_2_ molar flow; and V is the medium volume.

Equations VI, VII, VIII e IX were combined to obtain the initial equation used to update the inlet air flow (Q_air_) (Eq. X or Eq. B2.2, Box 2) and oxygen inlet fraction (y_O2,in_) (Eq. XI). In Eq. X, T_out_ is the outlet gas stream temperature and P_out_ is the outlet gas pressure.

$J_{O2}*C_{X}*V=\frac{Q_{O2,in}*P_{in}}{R*T_{in}}-\frac{y_{O2,out}*Q_{gas,out}*P_{out}}{R*T_{out}}$ (X)

$\text{y}_{\text{O2,in}}\text{=}\frac{\text{y}_{\text{O2,air}}\text{Q}_{\text{air}}\text{+}\text{y}_{\text{O2,N2}}\text{Q}_{\text{N2}}}{\text{Q}_{\text{air}}\text{+}\text{Q}_{\text{N2}}}$ (XI)

Equation XIV is obtained after grouping together few terms to set up the expressions $\text{α}$, $\text{β}_{\text{in}}$ and $\text{β}_{\text{out}}$, which are defined by Eqs. (XII), (XIIIa) and (XIIIb), respectively, and make the algebraic manipulations easier.

$\text{α}\text{=}\text{J}_{\text{O2}}\text{*}\text{C}_{\text{X}}\text{*}\text{V}$ (XII)

$\text{β}_{\text{in}}\text{=}\frac{\text{P}_{\text{in}}}{\text{R*}\text{T}_{\text{in}}}$ (XIIIa)

$\text{β}_{\text{out}}\text{=}\frac{\text{P}_{\text{out}}}{\text{R}\text{*}\text{T}_{\text{out}}}$ (XIIIb)

$\alpha=\text{(}\text{y}_{\text{O2,N2}}*\text{Q}_{\text{N2}}\text{+}\text{y}_{\text{O2,Air}}*\text{Q}_{\text{Air}})*\beta_{\mathrm{in}}-\text{y}_{\text{O2,out}}*\text{Q}_{\text{gas,out}}*\beta_{\mathrm{out}}$ (XIV)

The molar balance for nitrogen allows the calculation of the outlet gas flow rate ($\text{Q}_{\text{gas,out}}$) term (Eq. XV).

$\text{Q}_{\text{gas,out}}=\frac{\text{y}_{\text{N2,N2}}*\text{Q}_{\text{N2}}\text{+}\text{y}_{\text{N2,Air}}*\text{Q}_{\text{Air}}}{\text{y}_{\text{N}\text{2,out}}}$ (XV)

$\text{y}_{\text{N}\text{2,out}}=(1-\text{y}_{\text{O}\text{2,out}}-\text{y}_{\text{CO}\text{2,out}})$ (XVI)

Regarding Eq. XV, y_N2,out_ is the nitrogen fraction in the outlet gas stream; y_O2,out_ is the oxygen fraction in the outlet gas stream; and y_CO2,out_ is the carbon dioxide fraction in the outlet gas stream.

By combining Eq. XIV and XV, it is possible to isolate and obtain the final inlet air flow equation (Eq. XVII), after some manipulation.

$\alpha=\text{(}\text{y}_{\text{O2,N2}}*\text{Q}_{\text{N2}}\text{+}\text{y}_{\text{O2,Air}}*\text{Q}_{\text{Air}})*\beta_{in}-\text{y}_{\text{O2,out}}*\frac{\text{y}_{\text{N2,N2}}*\text{Q}_{\text{N2}}\text{+}\text{y}_{\text{N2,Air}}*\text{Q}_{\text{Air}}}{\text{y}_{\text{N}\text{2,out}}}*\beta_{out}$

$\text{γ}_{\text{out}}\text{=}\frac{\text{y}_{\text{O2,out}}}{\text{y}_{\text{N}\text{2,out}}}*\beta_{\mathrm{out}}$

$\alpha=\text{(}\text{y}_{\text{O2,N2}}*\text{Q}_{\text{N2}}\text{+}\text{y}_{\text{O2,Air}}*\text{Q}_{\text{Air}})*\beta_{\mathrm{in}}-(\text{y}_{\text{N2,N2}}*\text{Q}_{\text{N2}}\text{ }\text{+}\text{ }\text{y}_{\text{N2,Air}}*\text{Q}_{\text{Air}})*\text{γ}_{\text{out}}$

$\frac{\alpha}{\text{γ}_{\text{out}}}=\text{(}\text{y}_{\text{O2,N2}}*\text{Q}_{\text{N2}}\text{+}\text{y}_{\text{O2,Air}}*\text{Q}_{\text{Air}})*\frac{\beta_{\mathrm{in}}}{\text{γ}_{\text{out}}}-\text{y}_{\text{N2,N2}}*\text{Q}_{\text{N2}} \text{-}\text{ }\text{y}_{\text{N2,Air}}*\text{Q}_{\text{Air}}$

$\text{Q}_{\text{Air}}=\frac{\left( \frac{\alpha}{\text{γ}_{\text{out}}}\text{+}\text{y}_{\text{N2,N2}}*\text{Q}_{\text{N2}}\text{ - }\text{y}_{\text{O2,N2}}*\text{Q}_{\text{N2}}*\frac{\beta_{\mathrm{in}}}{\text{γ}_{\text{out}}} \right)}{\left( \frac{\beta_{\mathrm{in}}}{\text{γ}_{\text{out}}}*\text{y}_{\text{O2,Air}}-\text{y}_{\text{N2,Air}} \right)}$

$\text{Q}_{\text{Air}}=\frac{\left( \alpha\text{+}\text{y}_{\text{N2,N2}}*\text{Q}_{\text{N2}}\text{*}\text{γ}_{\text{out}}\text{ - }\text{y}_{\text{O2,N2}}*\text{Q}_{\text{N2}}*\beta_{\mathrm{in}} \right)}{\left( \text{y}_{\text{O2,Air}}*\beta_{\mathrm{in}}-\text{y}_{\text{N2,Air}}*\text{γ}_{\text{out}} \right)}$ (XVII)

Eq. (XVII) can be further reduced by assuming the oxygen fraction in the nitrogen gas stream ($\text{y}_{\text{O2,N2}}$) as zero ($\text{y}_{\text{O2,N2}}$ is below 0.01% and 0.001 % for industrial and ultrapure N_2_, respectively). Thus, equation XVIII is obtained, which can be further simplified if the oxygen fraction in the outlet stream ($\text{y}_{\text{O2,out}})$ is close to zero (what happens when the bioreactor is operated under severe oxygen limitation), yielding Eqs. (B2.2) and (B2.3), Box 2.

$\text{Q}_{\text{AIR}}\text{=}\frac{(Cx*J_{O2}^{\mathrm{MC}}*V*R)+Q_{N2}*\left( \frac{\text{y}_{\text{O2,out}}*\text{P}_{\text{out}}}{\text{y}_{\text{N2,out}}*\text{T}_{\text{out}}} \right)}{\left( \frac{\text{y}_{\text{O2,}\mathrm{air}}*\text{P}_{\mathrm{in}}}{\text{T}_{\mathrm{in}}} \right)-\text{y}_{\text{N2,}\mathrm{air}}*\left( \frac{\text{y}_{\text{O2,out}}*\text{P}_{\text{out}}}{\text{y}_{\text{N2,out}}*\text{T}_{\text{out}}} \right)}$ (XVIII)

Eqs. (B2.4) and (B2.5) (Box 2) are obtained from flux definition (Eq. 4, in the manuscript), where r_O2_ is given by Eq. (IX) and r_CO2_, analogously, is calculated using the component molar mass balance for CO_2_.
